# Gas-Phase Characterization of Adipic Acid, 6-Hydroxycaproic Acid, and Their Thermal Decomposition Products by Rotational Spectroscopy

**DOI:** 10.1021/acs.jpclett.3c02969

**Published:** 2024-01-17

**Authors:** Wenhao Sun, Pablo Pinacho, Daniel A. Obenchain, Melanie Schnell

**Affiliations:** † Deutsches Elektronen-Synchrotron DESY, Notkestr. 85, 22607 Hamburg, Germany; ‡ Institute of Physical Chemistry, Christian-Albrechts-Universität zu Kiel, Max-Eyth-Strasse 1, 24118 Kiel, Germany

## Abstract

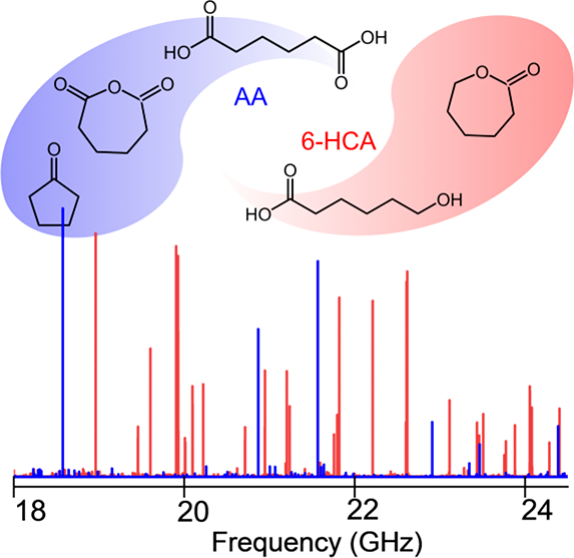

We report the spectroscopic investigation of two bifunctional aliphatic carboxylic acids, namely, adipic acid and 6-hydroxycaproic acid, in the gas phase by combining high-resolution rotational spectroscopy and supersonic expansions. Their pure rotational spectra were successfully identified and characterized. However, due to the low thermal stability of these two chemicals, the measured rotational spectra were significantly congested with transitions corresponding to their decomposition products upon heating. We observed cyclopentanone and adipic anhydride in the spectrum of adipic acid and *ε*-caprolactone and its monohydrate in the spectrum of 6-hydroxycaproic acid. On the basis of the distinct fingerprints of both carboxylic acids and a series of their decomposition products, the spectra were analyzed in a time-segmented manner. This provides valuable insights into the thermal decomposition mechanisms of these two samples over time, which highlights the robustness of microwave spectroscopy as a potent tool for analyzing complex chemical mixtures in a species-, isomer-, and conformer-selective way.

Adipic acid [HOOC-(CH_2_)_4_-COOH] and 6-hydroxycaproic acid [HOOC-(CH_2_)_5_-OH] are highly valuable six-carbon platform organic chemicals with significant industrial applications. Adipic acid (AA) is primarily used in the manufacturing of nylon-6,6 polyamide,^1^ one of the most popular synthetic fibers, and was regarded as the most important dicarboxylic acid in the polymer industry by the International Energy Agency in 2012.^2^ It also finds applications in the production of other materials, including plasticizers and polyurethanes, and in the food industry.^3,4^ On the contrary, 6-hydroxycaproic acid (6-HCA) and its dehydration product, *ε*-caprolactone (*ε*-CL), are mainly used as precursors to produce poly-*ε*-caprolactone (PCL).^5^*ε*-Caprolactone and PCL-based polymers are important biomaterials that are applied in various fields, such as tissue engineering and controlled drug delivery, owing to their controlled degradability and good biocompatibility.^6,7^ Given the wide applications of AA and 6-HCA/*ε*-CL, the industrial demand for them is quite high and continually increasing.^5,8^ The commercial manufacture of AA primarily relies on a popular two-step oxidation process of cyclohexane,^9,10^ wherein 6-HCA can be obtained as an intermediate species while producing AA.^11^ However, the overall product yield of the synthetic route is not yet satisfactory, and the reactions lead to significant emission of the potent greenhouse gas N_2_O into the atmosphere.^12^ Chemists have been devoting tremendous effort in the laboratory and industrial plants to developing more efficient and more environmentally friendly processes for the production of AA and its derivatives over the past several decades.^10,13−15^

The spectroscopic characterization of AA, 6-HCA, and the associated reaction intermediates during the synthesis processes, such as cyclohexanone, cyclohexanol, cyclohexyl hydroperoxide, and *ε*-CL, holds particular interest in this respect. Such analyses could provide valuable information about the structural configurations of these molecules. Characterizing with detail the conformational landscape of such products is crucial for unveiling reaction mechanisms under different conditions. In addition, these molecular species are also frequently detected within atmospheric secondary organic aerosols (SOA). They are introduced into the atmosphere through emissions from both anthropogenic and natural sources^16^ or generated within SOA as a result of photo-oxidation and ozonolysis processes involving cyclohexane and its derivatives.^15,17^ The characterization of these species would thus facilitate the understanding of the composition and chemistry of SOA. Traditionally, gas/liquid chromatography (GC/LC), commonly combined with mass spectrometry (MS), is employed to separate, identify, and quantify the composition of chemical mixtures.^18,19^ Various other techniques, including nuclear magnetic resonance (NMR) spectroscopy and X-ray crystallography,^15^ are applied for the chemical identification and characterization, as well.

In recent years, especially with the advent of broadband chirped-pulse Fourier transform microwave (CP-FTMW) spectroscopy,^20^ rotational spectroscopy has emerged as a novel and robust tool for chemical analysis.^21^ In microwave spectroscopy, rotational transitions are sensitive to the rotational constants (*A*, *B*, and *C*), which are inversely proportional to the molecular moments of inertia along the principal axes (*I*_*a*_, *I*_*b*_, and *I*_*c*_).^22^ As a result, the rotational fingerprints of molecules are unique and distinctive. Even in cases in which molecules share structural similarity, such as diastereomers, structural isomers, conformers, and isotopologues, their unambiguous identification becomes achievable through rotational spectroscopy, a capability that is challenging for other techniques. Moreover, on the basis of spectral identification and assignment, microwave spectroscopy allows for rapid quantification of chemical components in complex mixtures and real-time monitoring of reaction processes, which has been commercialized as microwave rotational resonance (MRR) spectroscopy.^23−25^

While the microwave spectra of the aforementioned intermediate species have been previously reported,^26−31^ studying the main products, AA and 6-HCA, is challenging due to their low vapor pressure and thermal instability. In this study, we present detailed microwave spectroscopic characterization of AA and 6-HCA, providing their rotational fingerprints for future chemical analysis. Notably, the observed microwave spectra also exhibited intense transitions arising from the thermal decomposition products of AA and 6-HCA. By employing theoretical methods, we explored the primary decomposition processes of these compounds, obtaining valuable insights into their stability upon heating.

The experiments were performed with our segmented CP-FTMW K-band spectrometer operating in the frequency range of 18–26 GHz. The instrument details have been described elsewhere.^32^ Commercially available samples of AA (99% purity) and 6-HCA (95% purity) were purchased from Sigma-Aldrich and used without further purification. Both of them appear as crystalline solids at room temperature with melting points of 152 and 38–40 °C, respectively. Their boiling points are 338 °C (AA) and 113–116 °C (6-HCA). The samples were individually placed into an internal reservoir, which is part of a custom-made stainless-steel nozzle adapted from a General Valve (Series 9). Through careful temperature testing, the samples of AA and 6-HCA were heated to 160 and 140 °C, respectively, achieving a balance between the vaporization and thermal decomposition processes. Neon (∼3 bar) was used as the carrier gas to deliver the diluted sample vapor into the vacuum chamber of the spectrometer via supersonic jet expansions in pulsed mode (10 Hz). The ensemble of molecules in each gas pulse was polarized with three microwave-pulse trains, containing ten chirp-pulse segments of 800 MHz covering the frequency range of 18–26 GHz. The effective repetition rate of the experiment is thereby 30 Hz. The free induction decay (FID) of the induced macroscopic polarization was recorded in the time domain for 10 μs after each excitation. The average of every 10^5^ FID acquisitions was saved, enabling the investigation of the spectral evolution as a function of the time segment (∼56 min), which is exploited below. In the experiments, approximately 2 × 10^6^ FIDs were collected in total for each compound. They are averaged and fast Fourier transformed to obtain the spectrum in the frequency domain. The spectral resolution is 100 kHz with a frequency accuracy of 20 kHz, providing a typical full width at half-maximum (FWHM) line width of ∼200 kHz when using the Kaiser–Bessel window function.

The experimental spectra were interpreted with the support of quantum-chemical calculations. The torsional degrees of freedom of the linear aliphatic chains [-(CH_2_)_4, 5_-] are 3^4^ and 3^5^ in AA and 6-HCA, respectively, leading to large ensembles of conformers. Thus, the conformational searches were carried out using the GFN-xTB method with the CREST routine. In total, 92 and 268 preliminary conformational configurations were obtained for AA and 6-HCA, respectively, within a relative energy window of 25 kJ/mol, which were used for further higher-level reoptimizations.^33,34^ The reoptimizations were performed at the B3LYP/def2-QZVP level of theory^35,36^ in combination with Grimme’s D4 dispersion corrections^37^ using ORCA version 4.2.1.^38^ Harmonic frequency calculations were performed, as well, to confirm that the energy minimum geometries are real and to provide zero-point energy (ZPE) corrections. The rotational constants and electric dipole-moment components were computed for the optimized minimum structures. In the following, the spectroscopic and theoretical results are described for the different components.

According to the quantum-chemical calculations for AA, there are nine conformers within a relative energy window of 5 kJ/mol. The geometries of the four most stable conformers are shown in Figure 1a, and their spectroscopic parameters are listed in Table 1. Additional information regarding the five remaining conformers can be found in Figure S1 and Table S1. Among them, AA-I and AA-IV possess a center of symmetry and thereby have no permanent dipole moments. Their rotational transitions are forbidden by the selection rules, so that they are absent from the spectrum, leaving AA-II and AA-III as the candidates to be observed. The rotational spectrum measured with the AA sample is displayed in Figure 2, in which we successfully identified a rotational fingerprint corresponding to AA. The observed transition frequencies were fit using Watson’s *S*-reduced Hamiltonian in the *I*^*r*^ representation with Pickett’s SPFIT program, as the molecule adopts a near-prolate rotor configuration.^39^ Note that other molecular species in this paper, which are more asymmetric, follow the same fitting procedure but with Watson’s *A*-reduced Hamiltonian. The experimentally determined rotational constants are consistent with those of AA-III, as compared in Table 1. The complete experimental spectroscopic parameters are listed in Table S3. The absence of AA-II is likely due to its relatively low electric dipole-moment components and the end-to-end structural arrangement that may facilitate its cyclization. There is also no evidence indicating the presence of higher-energy conformers.

**Figure 1:**
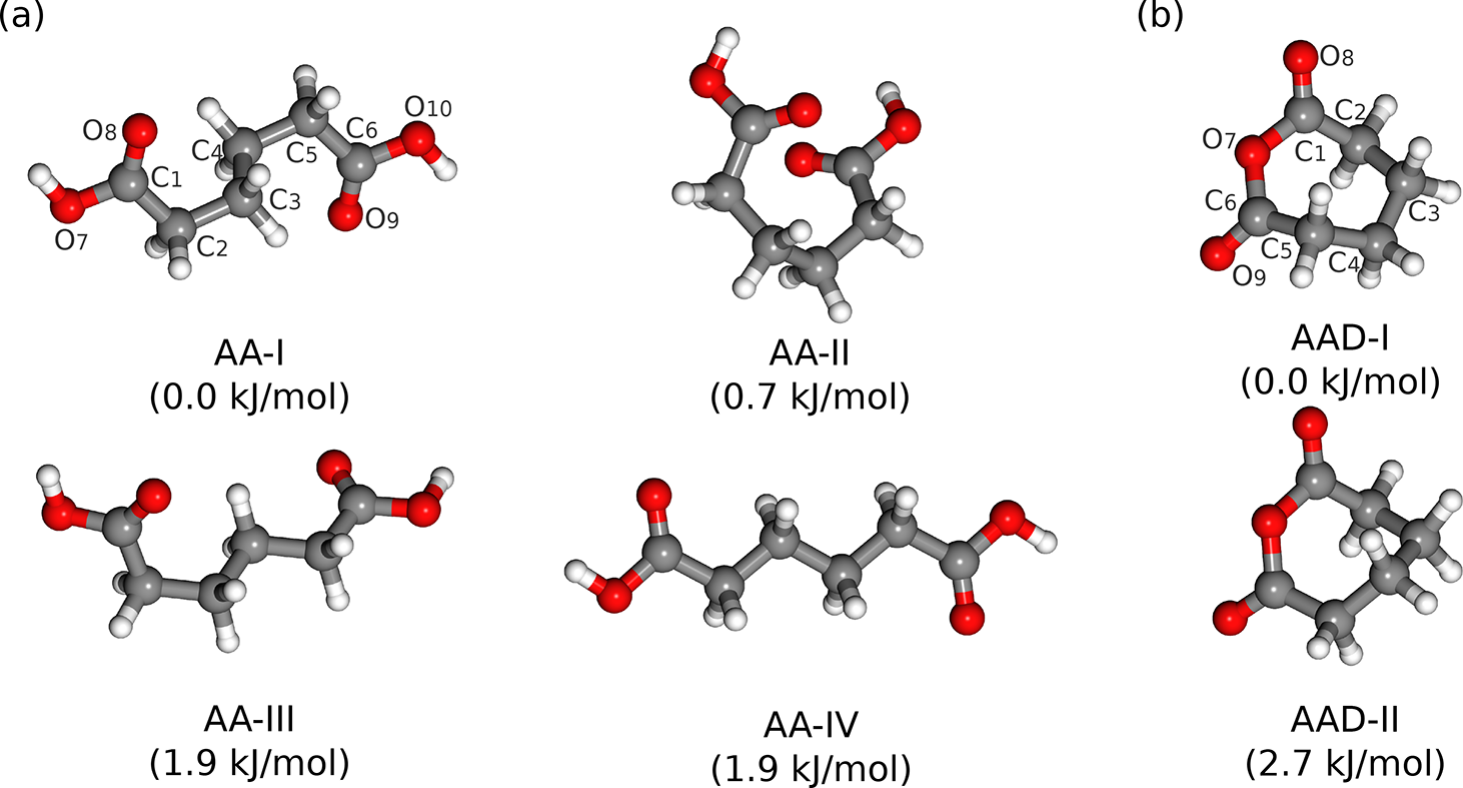
Molecular geometries of the main energy minima of (a) adipic acid (AA) and (b) adipic anhydride (AAD) within an energy window of 2 kJ/mol, optimized at the B3LYP-D4/def2-QZVP level of theory. The relative energies are corrected with vibrational zero-point energies, and those of AA-I and AAD-I are set as 0 kJ/mol.

**Figure 2:**
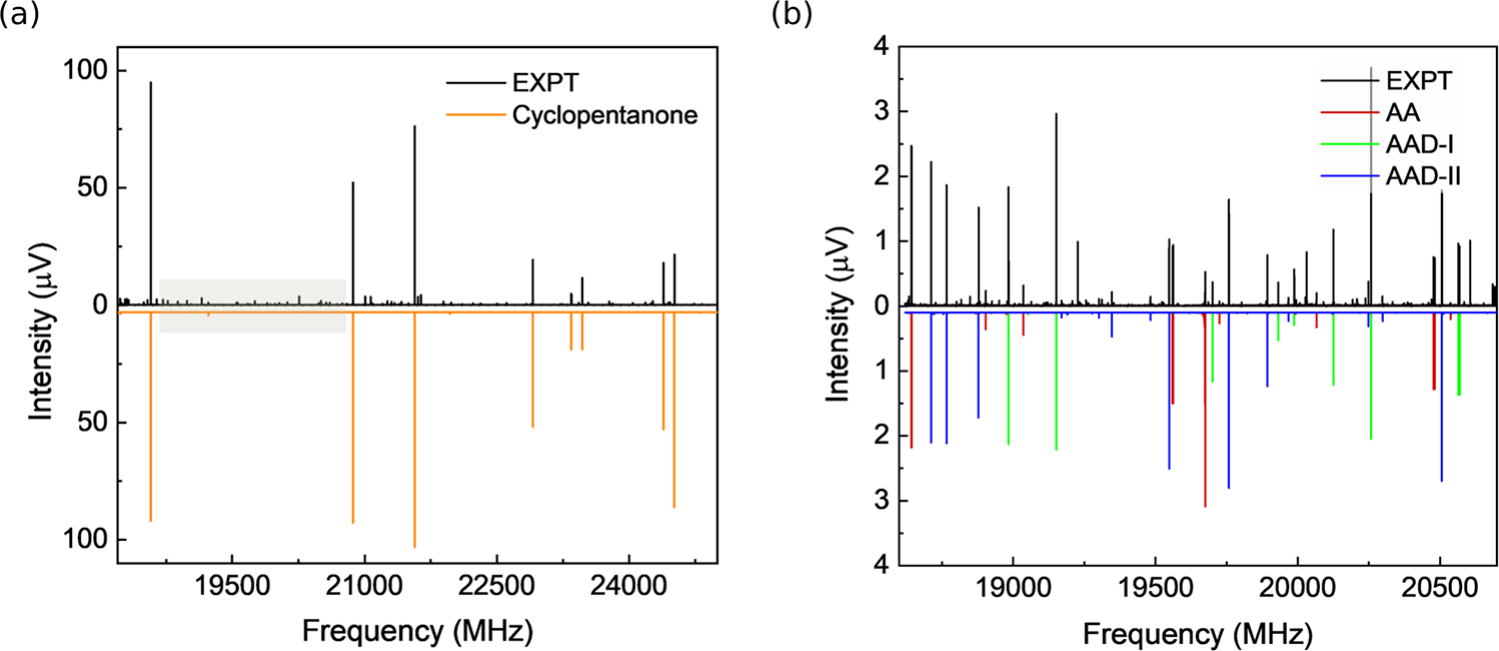
(a) Portion of the microwave spectrum measured with the adipic acid (AA) sample from 18.5 to 25 GHz. It consists of an average of 2.3 × 10^6^ FID acquisitions. The intense transitions arise from cyclopentanone, which is one of the thermal decomposition products from AA. (b) Magnified section of the spectrum from 18.6 to 20.7 GHz highlighting the rotational transitions arising from AA-III and its anhydrides, AAD-I and AAD-II. In both panels, the experimental spectrum (EXPT) is shown as the top trace, and the simulated spectra based on the fitted rotational constants of the identified molecular species are shown as the bottom trace.

**Table 1:** Theoretical and Experimental Spectroscopic Parameters for Adipic Acid (AA) and Adipic Anhydride (AAD)

	AA					AAD			
	theoretical^b^					theoretical^b^		experimental	
parameter^a^	I	II	III	IV	experimental	I	II	conformation I	conformation II
*A* (MHz)	4000	1655	3244	4963	3269.9551(32)	2173	2234	2170.70472(41)	2236.19273(44)
*B* (MHz)	480	1154	465	377	467.1814(11)	1727	1716	1739.00063(37)	1723.77081(34)
*C* (MHz)	452	848	449	354	450.6702(12)	1123	1157	1137.23992(38)	1173.99886(35)
|μ_*a*_| (D)	0.0	0.0	0.5	0.0	no	0.0	0.3	no	no
|μ_*b*_| (D)	0.0	0.7	1.8	0.0	yes	4.8	5.0	yes	yes
|μ_*c*_| (D)	0.0	0.0	0.5	0.0	yes	0.0	0.9	no	yes
*N*^c^					25			43	52
σ^d^ (kHz)					8.2			4.6	4.8
*ΔE*^e^ (kJ/mol)	0	0.7	1.9	1.9		0	2.7		

a The complete sets of spectroscopic parameters of AA and AAD are provided in Tables S3 and S4, respectively.

b Computed at the B3LYP-D4/def2-QZVP level of theory.

c Number of measured transitions.

d Root-mean-square deviations of the fit.

e Relative energy with vibrational zero-point energy corrections.

As shown in Figure 2a, the most intense transitions in the spectrum do not arise from AA. In fact, these lines can be attributed to one of its thermal decomposition products, cyclopentanone.^40,41^ Cyclopentanone is a colorless liquid at room temperature, with a boiling point of 131 °C. It has been known for more than a century as a decomposition product of AA (see eq 1),^42^ which is widely applied in the industrial production of cyclopentanone.^43,44^
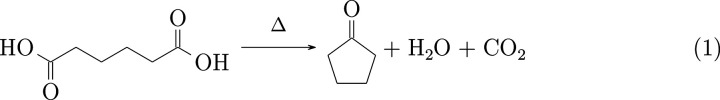
1The reaction is commonly catalyzed with substoichiometric amounts of bases [mainly Ba(OH)_2_] at temperatures from 250 to 450 °C, where AA undergoes a ketonic decarboxylation producing a near-stoichiometric amount of cyclopentanone.^44,45^ In this study, in the absence of catalytic bases, an intense spectrum of cyclopentanone is evidently observed at 160 °C, just on the verge of the reported decomposition temperatures (152–268 °C) of AA by a previous thermogravimetry-differential thermal analysis (TG-DTA) study, which observed a single mass-loss channel during the evaporation of AA.^46^ A variety of mechanisms have been proposed for the decomposition processes under different reaction conditions.^47^

For the linear dicarboxylic acids, their thermal decomposition behaviors are significantly influenced by the length of the aliphatic chain [-(CH_2_)*_n_*-] connecting the two carboxyl groups within the molecules, as demonstrated in eqs 1–5.^48^
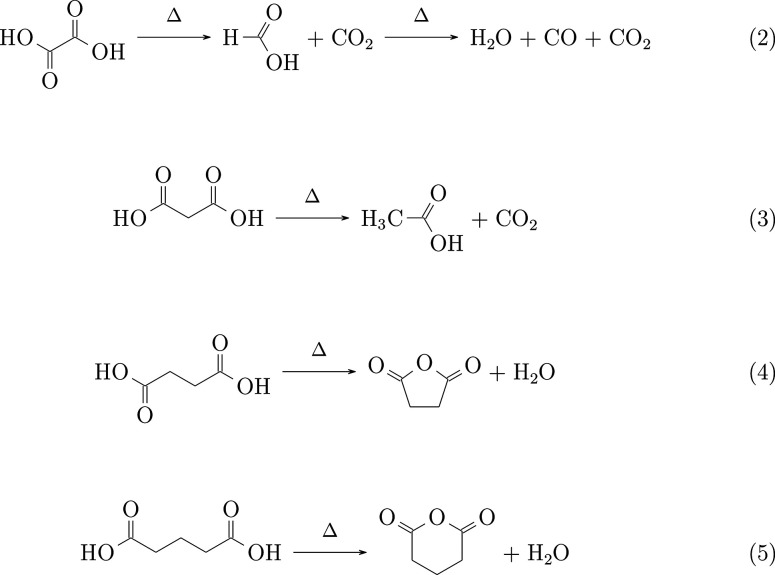
2In the case of oxalic acid (eq 2) and malonic acid (eq 3), the dominant decomposition reaction starts with the release of CO_2_, resulting in the formation of formic acid and acetic acid, respectively.^49,50^ As the number of the CH_2_ groups increases, succinic acid (eq 4) and glutaric acid (eq 5) tend to lose H_2_O instead of CO_2_, forming stable anhydrides under ambient conditions or mild heating. The rotational spectra of succinic acid and its anhydride have been investigated in detail previously.^51,52^ As the aliphatic chain length further extends, the decomposition process seems to follow another path, where the acids, such as adipic and pimelic acids (C_7_H_12_O_4_), decompose to CO_2_, H_2_O, and cyclic ketones with one fewer carbon. The observed intense spectrum of cyclopentanone in this study agrees with this decomposition pathway. The most accepted reaction mechanism proposes a ketonization process of the dicarboxylic acids via decarboxylation, which forms β-keto acids as intermediates instead of anhydrides.^45,47^ In this process, the two carboxyl groups tend to approach each other to adopt an arrangement similar to that in AA-II (see Figure 1),^47^ which also helps explain the absence of AA-II from the spectrum.

Alternatively, the ketonization reaction could also potentially take place on the basis of the formation of acid anhydrides, which are the main decomposition products for succinic acid and glutaric acid but have not yet been characterized for adipic acid.^53^ Throughout the measurement of AA, the experimental temperature (160 °C) exceeded the boiling point of water. The complex vapor mixture was continually delivered to the spectrometer via gas jets. As the spectral data were averaged and recorded for every 10^5^ FID acquisitions, this allows us to track changes in spectral intensity over the time segment. As shown in Figure 3, the spectral intensity of the water dimer at 24640.81 MHz, arising from the 2 0 *E*^+^–1 0 *E*^–^ rotational transition,^54^ gradually decreased, indicating the removal of water from the vapor mixture. The dry condition in the nozzle reservoir facilitates the formation of adipic anhydride (AAD).

**Figure 3:**
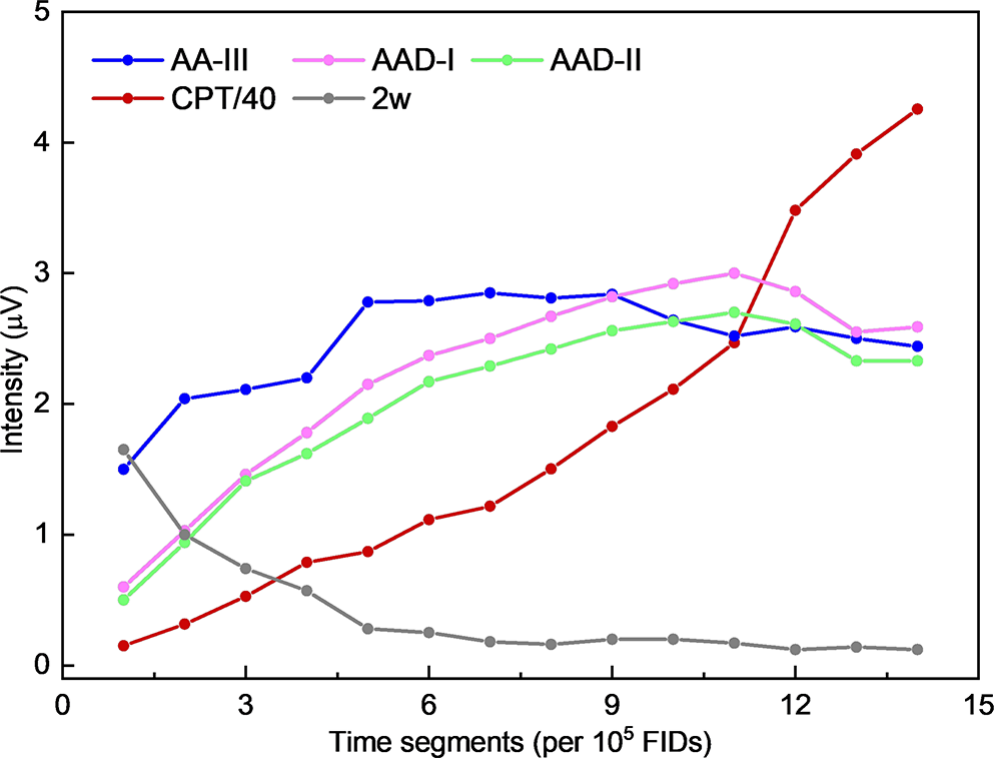
Line intensities of the rotational transitions arising from adipic acid (AA-III), the two conformers of adipic anhydride (AAD-I and AAD-II), cyclopentanone (CPT), and water dimer (2w) as a function of time. The monitored rotational frequencies are 18643.17 MHz (AA-III), 18229.39 MHz (AAD-I), 18712.13 MHz (AAD-II), 18575.42 MHz (CPT), and 24640.81 MHz (2w). The line intensity of CPT at 18575.42 MHz is scaled by a factor of 1/40 for the purpose of comparison.

In the same rotational spectrum, two conformers of AAD were unambiguously identified (Figure 2b), providing the first rotational spectroscopic characterization of AAD. Figure 1b shows that the seven-membered rings in the two most stable conformers of AAD adopt the chair (AAD-I) and twist-boat (AAD-II) configurations, respectively, compared to the prototype seven-membered ring of cycloheptane.^55^ The two conformations differ in energy by 2.7 kJ/mol. The corresponding spectroscopic parameters are listed in Table 1 and Table S4. Both spectra of AAD-I and AAD-II exhibit strengths comparable to those of AA-III. The intensities of rotational transitions are proportional to the population of the molecular species and the square of the magnitude of the associated transition dipole-moment components. In the spectra of AA-III, AAD-I, and AAD-II, b-type rotational transitions were observed, with associated dipole-moment components calculated to be 1.8, 4.8, and 5.0 D, respectively. Thereby, the population of AAD-I and AAD-II can be estimated to be ∼10% of AA in the gas jets. Note that the concentration of the key β-keto-acid intermediate formed in the ketonic decarboxylation pathway of AA was estimated to be ∼1% through colorimetry.^53,56^

Upon the formation of AAD, the subsequent decarboxylation can irreversibly lead to the production of cyclopentanone. According to the analysis of the line intensities as a function of time (Figure 3), the signals from AAD and cyclopentanone show positive correlations, which is in agreement with this hypothesis. This process is exothermic with a reaction energy (*ΔG*) of approximately −118 kJ/mol at 433.15 K in vacuum, as investigated using the nudged elastic band method at the B3LYP-D4/def2-QZVP level of theory.^57^ The associated activation barrier is approximately 253 kJ/mol (see Figure S3). On the contrary, the decomposition processes of succinic anhydride and glutaric anhydride, which lead to the formation of CO_2_, CO, and ethylene and the formaiton of CO_2_ and cyclobutanone, respectively, are endothermic. As such, these two cyclic anhydrides stand as the predominant thermal decomposition products of succinic acid and glutaric acid, respectively, rather than undergoing further decomposition.^58^

6-Hydroxycaproic acid (6-HCA) is a six-carbon ω-hydroxy carboxylic acid that also exhibits remarkable potential in various scientific fields and industrial applications. As the molecule contains both the carboxyl group and the hydroxy group, it can degrade through inter- and intramolecular esterification reactions upon thermal heating. The unimolecular ring-closure self-reaction is described in eq 6. Unlike the decomposition of AA, the esterification reactions are reversible, leading to the production of *ε*-caprolactone (*ε*-CL) and poly-*ε*-caprolactone (PCL), which can undergo hydrolysis to regenerate 6-HCA.
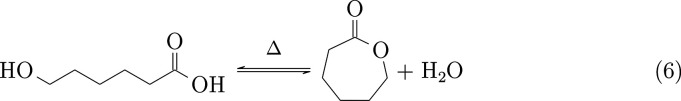
6

This classic reaction can proceed easily without additional catalysts. Previously, a variety of carboxylic acid–alcohol complex systems have been examined in the gas phase using microwave spectroscopy.^59^ By premixing primary/secondary alcohols with carboxylic acids (mainly formic acid) before the supersonic expansion, one observed solely the rotational spectra of the ester products. On the contrary, pre-reaction molecular complexes were observed instead in the spectra obtained with the tertiary alcohols and carboxylic acids. These results clearly suggest that for the formic acid–primary alcohol systems the esterification processes can be easily accessed when their vapors are mixed together. In 6-HCA, both primary alcohol and carboxyl groups are present, connected by five methylene groups. Figure 4a displays the three energetically low-lying conformers within an energy window of 5 kJ/mol. The geometries are mainly stabilized by intramolecular O–H···O hydrogen bonds between the terminal hydroxy group and carboxyl group, whereas the geometries of AA are mainly stabilized through the C–H···O hydrogen bonds between the aliphatic chain and the terminal carboxyl group. This structural arrangement of 6-HCA favors its ring-closure esterification reaction, posing challenges to the isolation of the monomeric 6-HCA in the gas phase. According to the theoretical calculations, the barrier of the esterification reaction pathway is ∼219 kJ/mol, yielding *ε*-CL and H_2_O with an energy release of ∼26 kJ/mol (see Table S10 and Figure S7).

**Figure 4:**
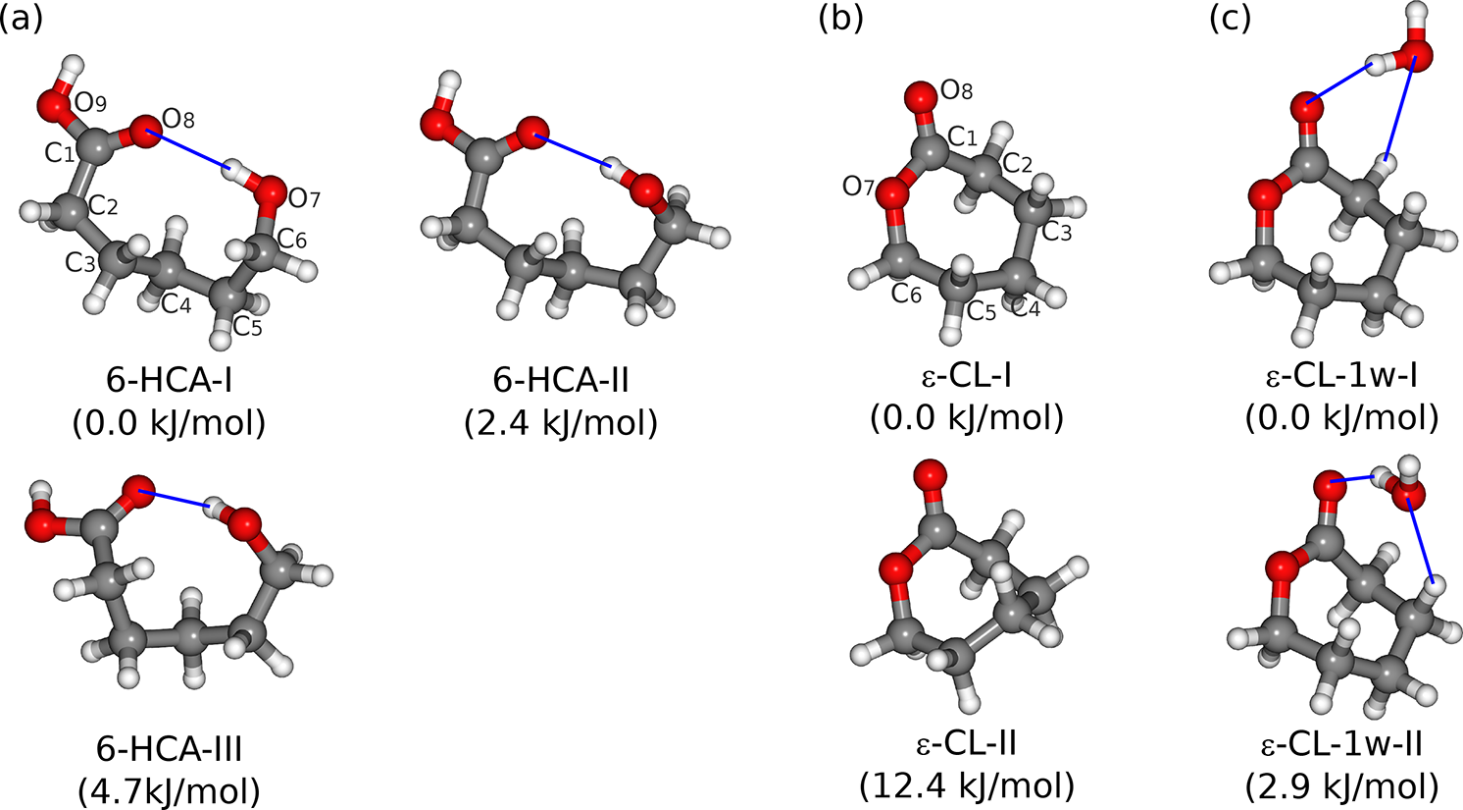
Molecular geometries of the main energy minima of (a) 6-hydroxycaproic acid (6-HCA), (b) *ε*-caprolactone (*ε*-CL), and (c) monohydrated *ε*-caprolactone (*ε*-CL–1w), optimized at the B3LYP-D4/def2-QZVP level of theory. The relative energies are corrected with vibrational zero-point energies, and those of 6-HCA-I, *ε*-CL-I, and *ε*-CL-1w-I are set as 0 kJ/mol. The blue lines indicate the O–H···O hydrogen bonds. Note that the energy difference between *ε*-CL-I and *ε*-CL-II was reported to be approximately 9 kJ/mol in ref (31) using MP2 and CCSD(T) methods.

As a cyclic ester, *ε*-CL possesses a seven-membered ring analogous to that of AAD (Figure 4b). The pure rotational spectrum has been reported with a comprehensive theoretical exploration of its conformational landscape.^30,31^ The two most stable conformers of *ε*-CL adopt the chair and twist-boat configurations, as in AAD-I and AAD-II, respectively, with both conformers identified previously.^31^ The energy difference is calculated to be approximately 9 kJ/mol using the MP2 and CCSD(T) methods in the previous theoretical study^31^ and 12.4 kJ/mol with the B3LYP-D4 method in this work. However, unlike AAD, which is readily converted into cyclopentanone, *ε*-CL exhibits remarkable thermal stability, making it a more preferred raw material than 6-HCA in the industrial production of PCL and PCL-based polymers. This divergent stability can be attributed to the ring strain inherent in the ester moieties, which can be described by two dihedral angles: C_6_–O_7_–C_1_–C_2_ and C_6_–O_7_–C_1_=O_8_ (Figure 4). In *ε*-CL-I and -II, these dihedral angles adopt values of −1° and 179° and 4.7° and 179°, respectively, signifying an almost planar configuration within the ester moiety for both conformations. In contrast, in AAD-I, characterized by *C*_2_ symmetry, the chemical environments of the two ester groups are identical, exhibiting dihedral angle counterparts of 37° and −147°. In AAD-II, the two ester groups display inequivalence (Figure 1b). For the upper ester group (O_7_–C_1_=O_8_), the dihedral angle counterparts are 53° and −133°; for the lower one (O_7_–C_6_=O_9_), these values are 8.1° and 172°. In both AAD conformations, the distortions of the two dihedral angles are substantially more pronounced than their counterparts in *ε*-CL-I and -II, resulting in the thermal instability observed in AAD and the high flexibility of the *ε*-CL ring, which favors its ring-opening reaction and polycondensation.^60^

Figure 5 presents the rotational spectrum measured with the 6-HCA sample, which is unsurprisingly dominated by the rotational transitions arising from *ε*-CL-I. The spectral intensities of the parent species of *ε*-CL-I allow for the observation of its ^13^C singly substituted isotopologues at their natural abundances (∼1.1%). Moreover, despite no addition of water, the molecular complex of *ε*-CL with one water molecule (*ε*-CL–1w) was identified in the spectrum, which can be considered as the product intermediate after the esterification reactions. Figure 4c shows the structures of the two energetically most stable isomers of the *ε*-CL–1w cluster. Both are formed with the global minimum conformation of monomer *ε*-CL-I. The water hydrogen atom prefers to bind with the carboxyl oxygen atom (O_8_) instead of the ester oxygen atom (O_7_), whereas the water oxygen atom is hydrogen bonded with the α-hydrogen in isomer I and β-hydrogen in isomer II. The energy difference between them is predicted to be 2.9 kJ/mol. Table 2 provides their theoretical spectroscopic constants. In total, 58 rotational transitions, including a-, b-, and c-type transitions, were observed in the spectrum of the water cluster. The experimentally determined rotational constants agree with those predicted for isomer I.

**Figure 5:**
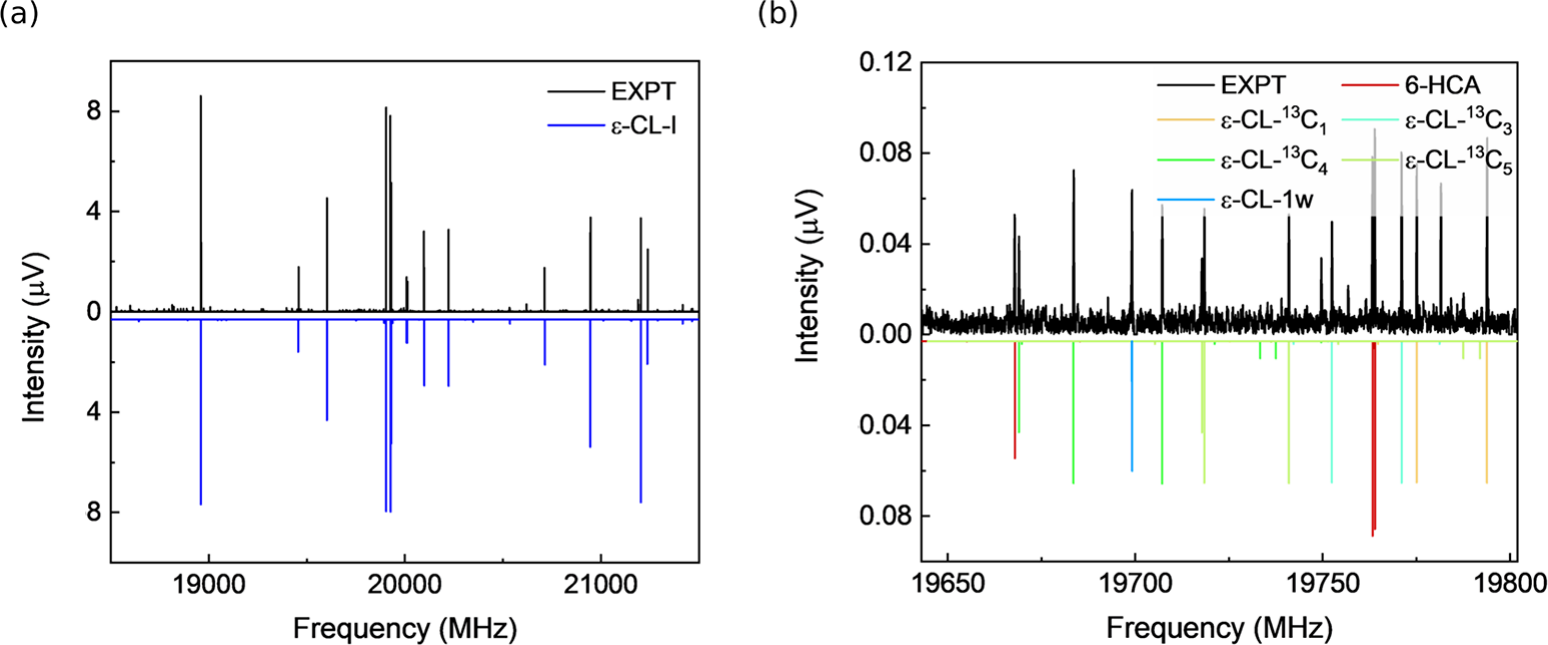
(a) Portion of the microwave spectrum measured with 6-hydroxycaproic acid (6-HCA) from 18.5 to 22 GHz. It was collected with 2.1 × 10^6^ FID acquisitions. The intense transitions arise from *ε*-caprolactone-I (*ε*-CL-I). (b) Magnified section of the spectrum from 19.6 to 19.8 GHz highlighting the transitions from 6-HCA, monohydrated *ε*-CL, and the monosubstituted ^13^C isotopologues of *ε*-CL-I. In both panels, the experimental spectrum (EXPT) is shown as the top trace, and the simulated spectra based on the fitted rotational constants of the assigned molecular species are shown as the bottom trace.

**Table 2:** Theoretical and Experimental Spectroscopic Parameters for the *ε*-Caprolactone–H_2_O (*ε*-CL–1w) Complex and 6-Hydroxycaproic Acid (6-HCA)

	*ε*-CL–1w			6-HCA			
	theoretical^b^			theoretical^b^			
parameter^a^	I	II	experimental	I	II	III	experimental
*A* (MHz)	2492	1790	2471.2105(15)	2669	2684	2289	2701.9420(35)
*B* (MHz)	1015	1244	1010.89618(58)	957	951	1092	939.0352(45)
*C* (MHz)	780	1151	777.06490(56)	765	783	907	757.7414(48)
|μ_*a*_| (D)	3.9	3.4	yes	2.3	2.1	2.2	yes
|μ_*b*_| (D)	1.1	0.2	yes	2.6	2.1	1.8	yes
|μ_*c*_| (D)	1.1	0.1	yes	0.5	1.2	1.6	no
*N*^c^			58				16
σ^d^ (kHz)			8.7				8.1
*ΔE*^e^ (kJ/mol)	0.0	2.9		0	2.4	4.7	

a The complete sets of spectroscopic parameters are provided in Tables S8 and S9.

b Computed at the B3LYP-D4/def2-QZVP level of theory.

c Number of measured transitions.

d Root-mean-square deviations of the fit.

e Relative energy with zero-point energy corrections.

With regard to 6-HCA, despite its moderate melting point (38–40 °C) and boiling point (113–116 °C), the spectrum was observed when the sample was heated to 140 °C during the experiment (see Figure S6). As shown in Figure 5b, the spectral strength appears slightly greater than that of the ^13^C monosubstituted isotopologues of *ε*-CL-I. A total of two a-type and 14 b-type rotational transitions were observed for 6-HCA, despite both dipole-moment components being predicted to be of the same magnitude (Table 2). The determined rotational constants closely match those predicted for conformers I and II. The main geometric difference between these two conformers lies in the conformation of the terminal hydroxymethyl group (-CH_2_-OH), leading to an energy discrepancy of 2.4 kJ/mol and a more pronounced μ_*c*_ electric dipole-moment component in 6-HCA-II than in 6-HCA-I. As there is no c-type transition evidenced in the spectrum, global-minimum conformer I is more likely the carrier of the observed rotational fingerprint. According to the time-segmented analysis (Figure S7), the spectral signatures of 6-HCA began to decline after the collection of 2 × 10^5^ FIDs (∼2 h), indicating a more rapid reaction in comparison to the decomposition of AA.

It is noteworthy that, similar to the size-dependent thermal behaviors observed in dicarboxylic acids, the thermodynamic properties of the hydroxy carboxylic acids, encompassing reactivity and thermal stability, are profoundly influenced by the size of the aliphatic chain bridging the hydroxy and carboxyl groups.^61^ Additionally, considering the esterification reaction as a reversible process, the stability of the hydroxy carboxylic acids is also intricately tied to the ring stability of their ensuing cyclic lactone products.^60^ Taking the three structural isomers of hydroxybutyric acid (C_4_H_8_O_3_) as an example, one observed the rotational spectra of 2- and 3-hydroxybutyric acids with minimal contributions from their decomposition products. On the contrary, upon examination of the spectra acquired from 4-hydroxybutyric acid, resembling 6-HCA but with two fewer methylene units, only γ-butyrolactone was detected.^62^ γ-Butyrolactone is known for its poor polymerizability in comparison with δ-valerolactone and *ε*-caprolactone. This contrast finds its explanation in the varying stability of the ring structures present in the cyclic lactones.^62^ Notably, the process of ring opening in γ-butyrolactone proves to be endergonic, in contrast to the exergonic nature observed in δ-valerolactone and *ε*-caprolactone. As a result, this endergonic characteristic would also hinder the hydrolysis of γ-butyrolactone, shifting the reaction equilibrium of the esterification process of 4-hydroxybutyric acid to the right side. In the case of 6-HCA, the increased flexibility of its seven-membered ring promotes its ring opening and subsequent hydrolysis, rationalizing the observed detection of 6-HCA in our experimental context.

In summary, we present the chirped-pulse Fourier transform microwave spectroscopic experiments conducted on two six-carbon bifunctional organic molecules, namely, adipic acid and 6-hydroxycaproic acid, in the gas phase, in combination with quantum-chemical calculations. During the experiments, a large portion of adipic acid underwent ketonization upon mild heating, while 6-hydroxycaproic acid underwent esterification. This leads to the formation of cyclopentanone and adipic anhydride for adipic acid and *ε*-caprolactone for 6-hydroxycaproic acid. Consequently, the experimental spectra were dominated by these decomposition products. By carefully adjusting the temperature during the experiments, we successfully identified the rotational spectra arising from adipic acid and 6-hydroxycaproic acid, thus providing their rotational spectroscopic characterizations for the first time. These two experiments not only shed light on the vaporization conditions of the two compounds but also offer valuable fingerprints for analyzing relevant industrial and scientific samples and monitoring their concentrations during the synthesis processes using microwave spectroscopy.

## Supporting Information Available

Theoretical calculations, experimental spectroscopic parameters, thermal decomposition pathways, Cartesian coordinates, and experimental transition frequencies for the studied molecular species, namely, adipic acid, adipic anhydride, 6-hydroxycaproic acid, and monohydrated *ε*-caprolactone (PDF)
