# Supplementary material for: Gas-Phase Characterization of Adipic Acid, 6-Hydroxycaproic Acid, and Their Thermal Decomposition Products by Rotational Spectroscopy
Source: J Phys Chem Lett. 2024 Jan 17;15(3):817–25. doi: 10.1021/acs.jpclett.3c02969 (PMC10823529; doi:10.1021/acs.jpclett.3c02969)
Supplement: Supplementary file 1 — jz3c02969_si_001.pdf [file jz3c02969_si_001.pdf]

Supporting information:

Gas-Phase Characterization of Adipic Acid, 6-Hydroxycaproic Acid, and Their Thermal Decomposition Products by Rotational Spectroscopy

Wenhao Sun,[*,†] Pablo Pinacho,[†,¶] Daniel A. Obenchain,[†,§] and Melanie Schnell[*,†,‡]

†*Deutsches Elektronen-Synchrotron DESY, Notkestr. 85, 22607 Hamburg, Germany*

‡*Institute of Physical Chemistry, Christian-Albrechts-Universität zu Kiel, Max-Eyth-Str. 1, 24118 Kiel, Germany*

¶*Current address: Department of Physical Chemistry, University of the Basque Country (UPV/EHU), B° Sarriena, S/N, Leioa, 48940, Spain*

§*Current address: Georg-August-Universität Göttingen, Institut für Physikalische Chemie, 37077 Göttingen, Germany*

E-mail: wenhao.sun@desy.de; melanie.schnell@desy.de

Contents

1 Adipic acid. **S3**

1.1 Theoretical predictions of the conformers of adipic acid. S3

1.2 Theoretical predictions of the conformers of adipic anhydride. S4

1.3 Experimental assignments. S5

1.4 Decarboxylation pathways of adipic anhydride. S6

2 6-Hydroxycaproic acid. **S8**

2.1 Theoretical predictions of the conformers of 6-hydroxycaproic acid. S8

2.2 Theoretical predictions of the conformers of the monohydrated ε-caprolactone. S8

2.3 Experimental assignments. S9

2.4 Measurements performed at different temperatures. S11

2.5 Time-segmented analysis of the 6-HCA microwave spectrum. S12

2.6 Esterification reaction pathway of 6-hydroxycaproic acid. S12

3 Cartesian coordinates of molecular geometries. **S14**

3.1 Conformers of adipic acid. S14

3.2 Conformers of adipic anhydride. S22

3.3 Conformers of ε-caprolactone. S24

3.4 Isomers of the monohydrated ε-caprolactone. S26

3.5 Conformers of the 6-hydroxycaproic acid. S28

4 Measured rotational transitions. **S31**

4.1 Frequency list of adipic acid. S31

4.2 Frequency list of adipic anhydride. S33

4.3 Frequency list of the monohydrated ε-caprolactone. S37

4.4 Frequency list of 6-hydroxycaproic acid. S42

1 Adipic acid.

1.1 Theoretical predictions of the conformers of adipic acid.

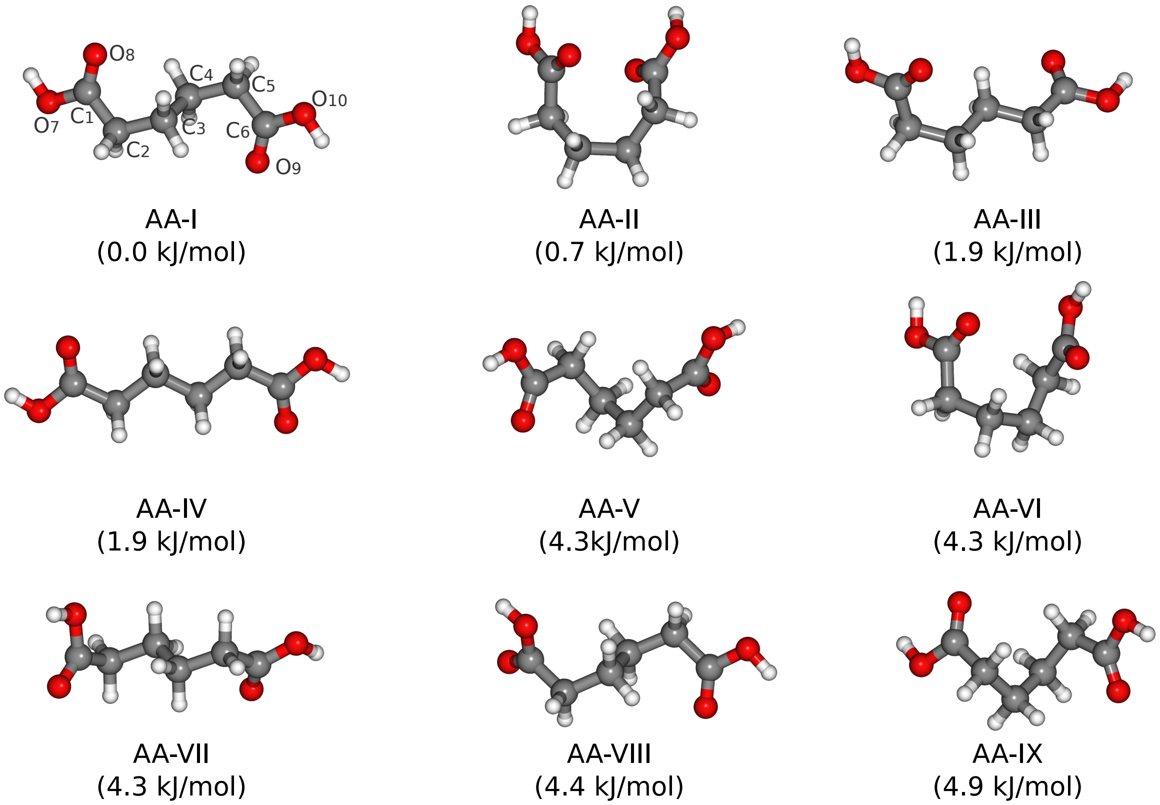

Figure S1: Molecular geometries of the energetically low-lying conformers of adipic acid (AA) within an energy window of 5 kJ/mol, calculated at the B3LYP-D4/def2-QZVP level of theory. The relative energies are corrected with zero-point energies and that of AA-I is set to 0 kJ/mol. The AA-I structure shows the atom labelling. The corresponding Cartesian coordinates are available in Tables S11–S19 in Section 3.

Table S1: Theoretical spectroscopic constants for the conformers of adipic acid (AA), as provided in Figure S1, calculated at the B3LYP-D4/def2-QZVP level of theory.

Conformers	ΔE_{ZPE}/kJ mol^{-1}	A/MHz	B/MHz	C/MHz	μ_a/D	μ_b/D	μ_c/D
AA-I	0.0	4000	480	452	0.0	0.0	0.0

Conformers	ΔE_{ZPE}/kJ mol^{-1}	A/MHz	B/MHz	C/MHz	μ_a/D	μ_b/D	μ_c/D
AA-II	0.7	1655	1154	848	0.0	0.7	0.0
AA-III	1.9	3244	465	449	-0.5	-1.8	0.5
AA-IV	1.9	4963	377	354	0.0	0.0	0.0
AA-V	4.3	2709	596	554	0.0	-2.1	0.0
AA-VI	4.3	1847	960	761	1.7	-0.3	-1.9
AA-VII	4.3	3569	494	479	1.2	2.0	-1.2
AA-VIII	4.4	3552	495	481	-1.2	-2.1	1.2
AA-IX	4.9	2745	645	592	-0.6	0.3	0.0

1.2 Theoretical predictions of the conformers of adipic anhydride.

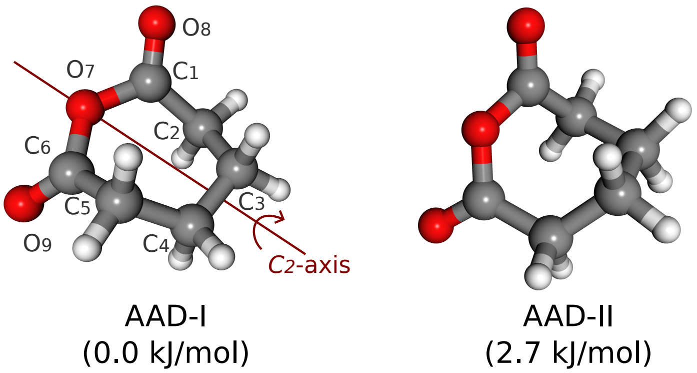

Figure S2: Molecular geometries of the two most energetically stable conformers of adipic anhydride (AAD), calculated at the B3LYP-D4/def2-QZVP level of theory. The relative energies are corrected with zero point energies. In the geometry of AAD-I, the C_2 axis of molecular symmetry is displayed. The corresponding Cartesian coordinates are available in Tables S20–S21 in Section 3.

Table S2: Theoretical spectroscopic constants for the conformers of adipic anhydride (AA), as provided in Figure S2, calculated at the B3LYP-D4/def2-QZVP level of theory.

Conformers	ΔE_{ZPE}/kJ mol^{-1}	A/MHz	B/MHz	C/MHz	μ_a/D	μ_b/D	μ_c/D
AAD-I	0.0	2173	1727	1123	0.0	4.8	0.0
AAD-II	2.7	2234	1716	1157	-0.3	-5.0	-0.9

1.3 Experimental assignments.

Table S3: Experimental spectroscopic constants for AA-III, fitted with Watson's S-reduction Hamiltonian in its I^r representation using the Pickett's SPFIT program.

Parameters	Unit	AA-III
A	MHz	3269.9551(32)
B	MHz	467.1814(11)
C	MHz	450.6702(12)
Δ_J	kHz	0.0575(34)
Δ_{JK}	kHz	-1.146(26)
Δ_K	kHz	12.73(13)
δ_J	kHz	0.0079(23)
δ_K	kHz	0.00066(31)
a-type		No
b-type		Yes
c-type		Yes
# of lines		31
RMS	kHz	8.2

Table S4: Experimental spectroscopic constants for AAD-I and AAD-II, fitted with Watson's A-reduction Hamiltonian in its I^r representation using the Pickett's SPFIT program.

Parameters	Unit	AAD-I	AAD-II
A	MHz	2170.70472(82)	2236.19273(44)
B	MHz	1739.00063(73)	1723.77081(34)
C	MHz	1137.23992(75)	1173.99886(35)
Δ_J	kHz	0.1410(65)	0.1924(32)
Δ_{JK}	kHz	2.292(22)	0.242(10)
Δ_K	kHz	-2.214(22)	0.083(14)
δ_J	kHz	-0.0021(23)	0.0028(11)
δ_K	kHz	1.236(18)	0.3950(92)
a-type		No	No
b-type		Yes	Yes
c-type		No	Yes
# of lines		43	52
RMS	kHz	4.6	4.8

1.4 Decarboxylation pathways of adipic anhydride.

Table S5: Enthalpy (H), entropy (S), and Gibbs free energies (G=H-T*S) of the reactants (AAD-I and AAD-II), transition states (TS-I and TS-II), and products (CO_2+cyclopentanone) at 433.15 K, during the decarboxylation processes of adipic anhydride (AAD), calculated at the B3LYP-D4/def2-QZVP level of theory.

	Enthalpy (H) /Hartree	Entropy (S) /Hartree	Gibbs free energy (G) /Hartree	ΔG (T= 433.15 K) kJ/mol
AAD-I	-458.986266	-0.000139	-459.046569	0.0

	Enthalpy (H)	Entropy (S)	Gibbs free energy (G)	ΔG (T= 433.15 K)
TS-I	-458.889528	-0.000140	-458.950384	252.7
CO_2+CPT	-459.0050239	-0.000200	-459.091703	-118.4
AAD-II	-458.9852911	-0.000139	-459.0554791	0.0
TS-II	-458.8905407	-0.000140	-458.951163	274.1
CO_2+CPT	-459.0050239	-0.000200	-459.091703	-95.0

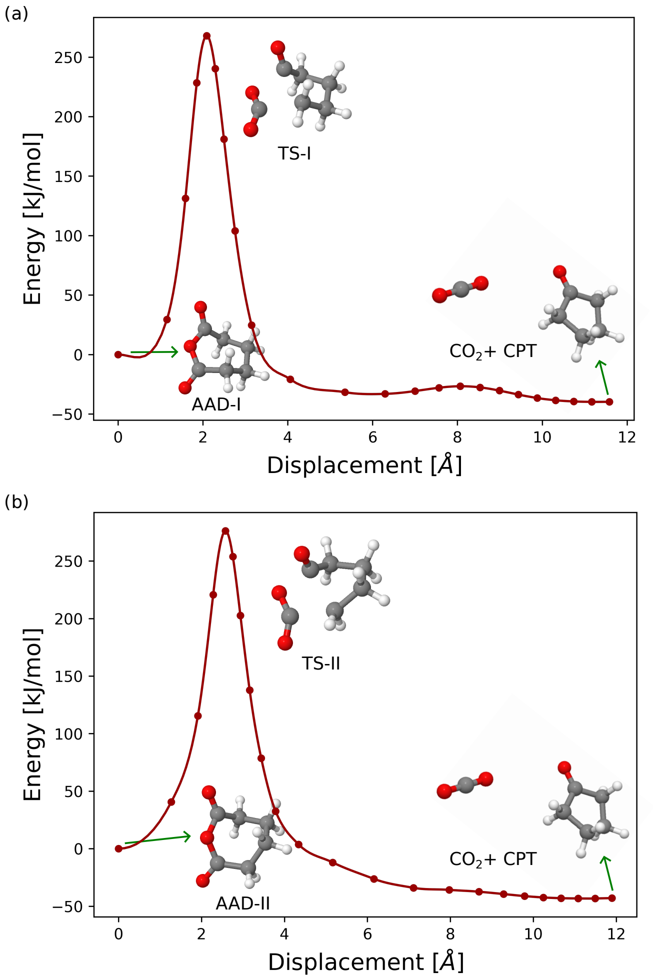

Figure S3: Decarboxylation reaction pathways of AAD-I (a) and AAD-II (b) to the products of CO_2 + cyclopentanone (CPT), calculated through the nudged elastic band (NEB) method[1] at the B3LYP-D4/def2-QZVP level of theory using the ORCA 4.2.1 program package.

2 6-Hydroxycaproic acid.

2.1 Theoretical predictions of the conformers of 6-hydroxycaproic acid.

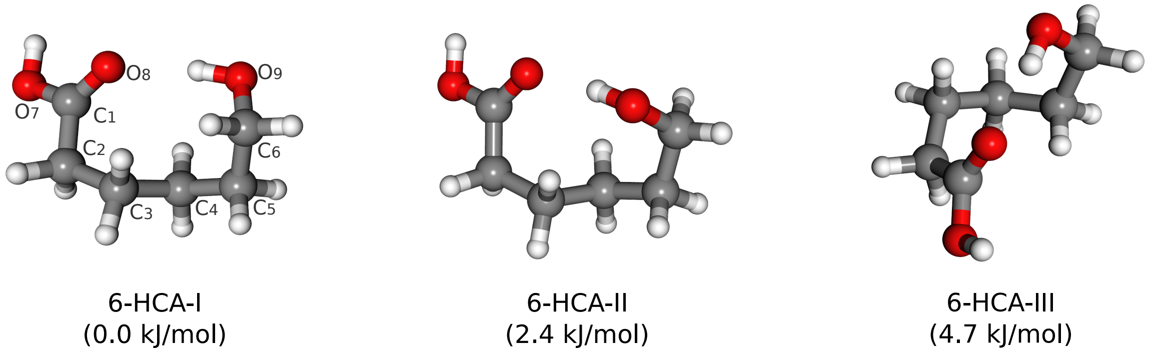

Figure S4: Molecular geometries of the energetically low-lying conformers of 6-hydroxycaproic acid (6-HCA) within an energy window of 5 kJ/mol, calculated at the B3LYP-D4/def2-QZVP level of theory. The relative energies are corrected with zero-point energies and that of 6-HCA-I is set to 0 kJ/mol. The corresponding Cartesian coordinates are available in Tables S26–S28 in Section 3.

Table S6: Theoretical spectroscopic constants for the conformers of 6-hydroxycaproic acid (6-HCA), as provided in Figure S4, calculated at the B3LYP-D4/def2-QZVP level of theory.

Conformers	ΔE_{ZPE}/kJ mol^{-1}	A/MHz	B/MHz	C/MHz	μ_a/D	μ_b/D	μ_c/D
6-HCA-I	0	2669	957	766	2.3	2.6	-0.5
6-HCA-II	2.4	2684	951	783	2.1	-2.1	1.2
6-HCA-III	4.7	2289	1092	907	2.2	1.8	-1.6

2.2 Theoretical predictions of the conformers of the monohydrated ε-caprolactone.

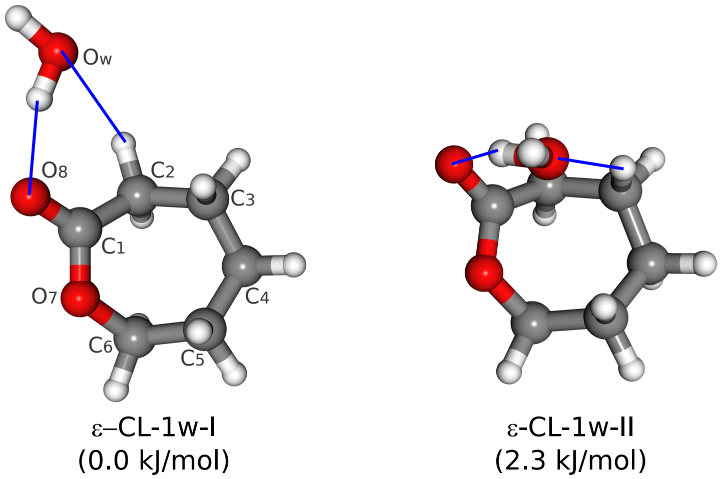

Figure S5: Molecular geometries of the energetically low-lying conformers of the monohydrated ε-caprolactone (ε-CL-1w) within an energy window of 5 kJ/mol, calculated at the B3LYP-D4/def2-QZVP level of theory. The relative energies are corrected with zero-point energies and that of ε-CL-1w-I is set to 0 kJ/mol. The corresponding Cartesian coordinates are available in Tables S24–S25 in Section 3.

Table S7: Theoretical spectroscopic constants for the two isomers of the monohydrated ε-caprolactone (ε-CL-1w), as provided in Figure S4, calculated at the B3LYP-D4/def2-QZVP level of theory.

Isomers	ΔE_{ZPE}/kJ mol^{-1}	A/MHz	B/MHz	C/MHz	μ_a/D	μ_b/D	μ_c/D
ε-CL-1w-I	0	2492	1015	780	3.9	1.1	1.1
ε-CL-1w-II	2.9	1790	1244	1151	3.4	0.2	0.1

2.3 Experimental assignments.

Table S8: Experimental spectroscopic constants for 6-hydroxycaproic acid (6-HCA-I), fitted with Watson's A-reduction Hamiltonian in its I^r representation using the Pickett's SPFIT program.

Parameters	Unit	6-HCA-I
A	MHz	2701.9420(24)

Parameters	Unit	6-HCA-I
B	MHz	939.0352(31)
C	MHz	757.7414(33)
Δ_J	kHz	0.194(13)
Δ_{JK}	kHz	-0.985(31)
Δ_K	kHz	3.229(87)
δ_J	kHz	0.074(10)
a-type		Yes
b-type		Yes
c-type		No
# of lines		16
RMS	kHz	5.2

Table S9: Experimental spectroscopic constants for the monohydrated ε-caprolactone (ε-CL-1w), fitted with Watson's A-reduction Hamiltonian in its I^r representation using the Pickett's SPFIT program. The spectrum of ε-CL-$H_2{}^{18}O$ was measured separately with the addition of ^{18}O enriched water.

Parameters	Unit	ε-CL-$H_2{}^{16}O$	ε-CL-$H_2{}^{18}O$
A	MHz	2471.2105(15)	2459.7398(14)
B	MHz	1010.89618(58)	962.09751(38)
C	MHz	777.06490(56)	746.93902(31)
Δ_J	kHz	2.5241(22)	2.4470(10)
Δ_{JK}	kHz	-15.541(10)	-15.1038(64)
Δ_K	kHz	32.211(67)	33.209(62)
δ_J	kHz	0.66549(62)	0.62715(45)
δ_K	kHz	0.898(22)	1.203(13)
a-type		Yes	Yes
b-type		Yes	Yes

Parameters	Unit	ε-CL-$H_2{}^{16}O$	ε-CL-$H_2{}^{18}O$
c-type		No	No
# of lines		58	58
RMS	kHz	8.7	6.5

2.4 Measurements performed at different temperatures.

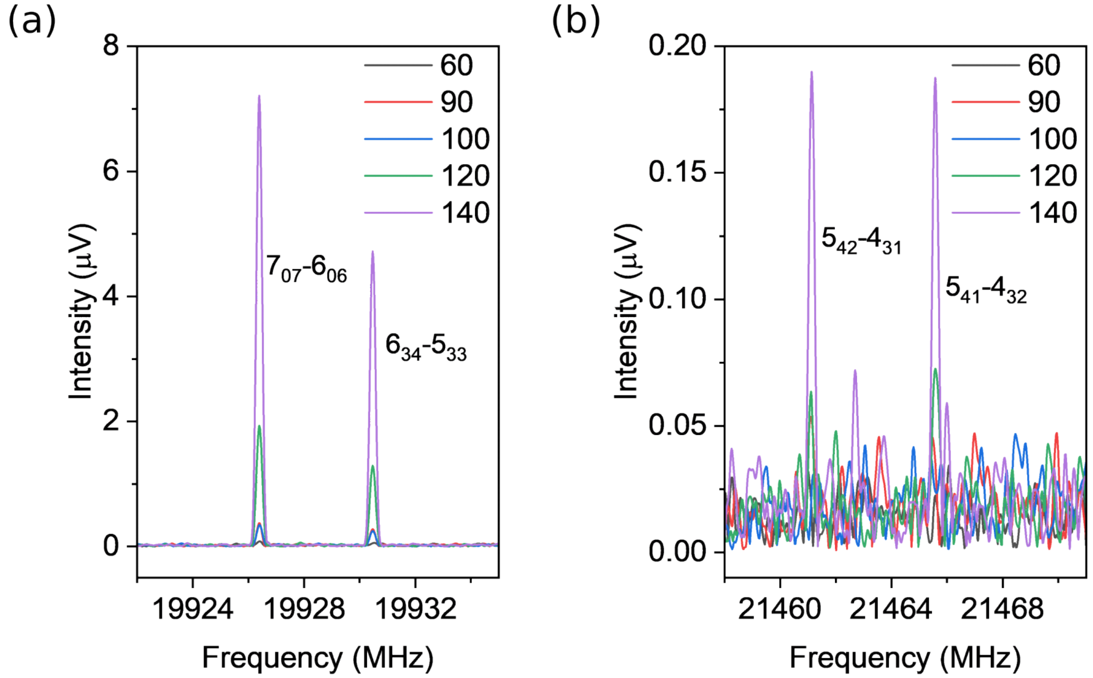

Figure S6: Rotational transitions of ε-caprolactone (a) and 6-hydroxycaproic acid (b), monitored at different sample temperatures (°C). Each spectrum was collected with 2×10^5 FID acquisitions. The rotational state are denoted as JK_aK_c, where J represents the total angular momentum, K_a and K_c are the projections of the angular momentum onto the principal molecular axes a and c. Note that measurements at 160 and 180 °C were also performed but not successful because the sample underwent rapid reactions, leading to the blockage of the nozzle orifice by decomposition products after a short period.

2.5 Time-segmented analysis of the 6-HCA microwave spectrum.

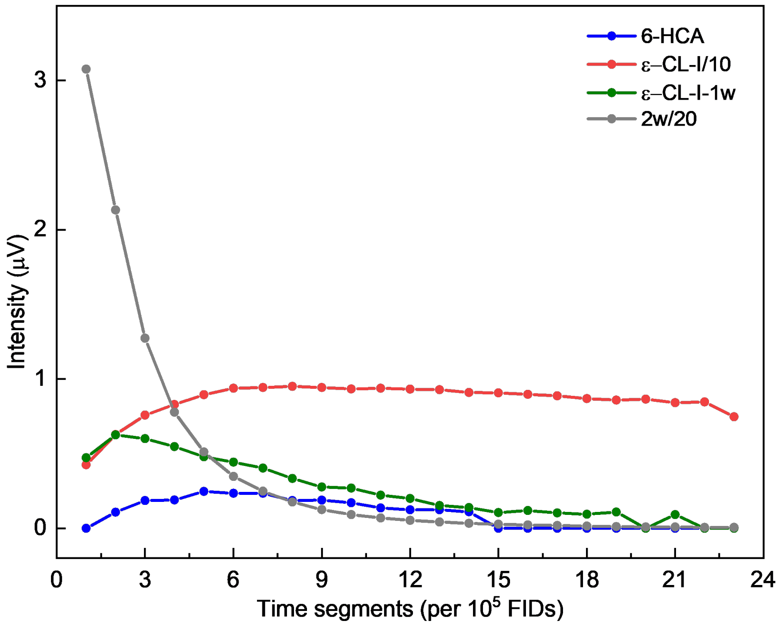

Figure S7: Line intensities of the rotational transitions arising from 6-hydroxycaproic acid (6-HCA), ε-caprolactone (ε-CL-I), and water dimer (2w) as a function of per 10^5 FID acquisitions. The monitored rotational frequencies are 21465.58 MHz (6-HCA), 18960.10 MHz (ε-CL-I), 18813.16 MHz (ε-CL-I-1w), and 24640.81 MHz (2w), respectively. The line intensities corresponding to ε-CL-I and 2w are scaled by a factor of 1/10 and 1/20, respectively.

2.6 Esterification reaction pathway of 6-hydroxycaproic acid.

Table S10: Enthalpy (H), entropy (S), and Gibbs free energies (G=H-T*S) of the 6-hydroxycaproic acid (6-HCA), transition states (TS), the monohydrated ε-caprolactone (ε-CL-1w-I), and the final products (H_2O + ε-CL) at 413.15 K, during the decarboxylation processes of 6-HCA, calculated at the B3LYP-D4/def2-QZVP level of theory.

	Enthalpy (H) /Hartree	Entropy (S) /Hartree	Gibbs free energy (G) /Hartree	ΔG (T= 433.15 K) kJ/mol
6-HCA	-461.352885	-0.000153	-461.415997	0.0
TS	-461.272522	-0.000145	-461.332587	218.8
ε-CL-1w-I	-461.351086	-0.000158	-461.416547	-1.3

	Enthalpy (H)	Entropy (S)	Gibbs free energy (G)	ΔG (T= 433.15 K)
H_2O + ε-CL-I	-461.341550	-0.000204	-461.425887	-25.9

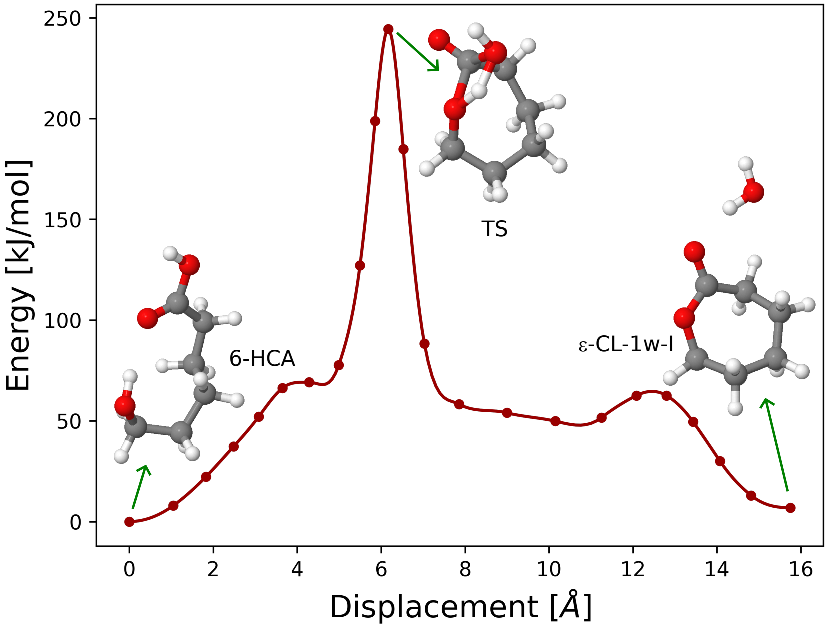

Figure S8: Esterification reaction pathway from the global minimum conformer of 6-hydroxycaproic acid (6-HCA-I) to the products of the monohydrated ε-caprolactone (ε-CL-1w-I), calculated through the nudged elastic band (NEB) method[1] at the B3LYP-D4/def2-QZVP level of theory using the ORCA 4.2.1 program package.

References

(1) Jónsson, H.; Mills, G.; Jacobsen, K. W. *Nudged Elastic Band Method for Finding Minimum Energy Paths of Transitions*; World Scientific, 1998; pp 385–404.

3 Cartesian coordinates of molecular geometries.

3.1 Conformers of adipic acid.

Table S11: Cartesian coordinates for the equilibrium structure of conformer I of adipic acid (AA-I) optimized at the B3LYP-D4/def2-QZVP level of theory.

Atom	X	Y	Z
C	-1.718733	-0.822448	-0.437395
C	-2.752890	0.220263	-0.092682
O	-2.691777	1.008154	0.814665
O	-3.817677	0.162234	-0.927963
H	-4.449805	0.831315	-0.630038
C	-0.379165	-0.611544	0.259226
H	-0.547891	-0.506729	1.332463
H	0.228302	-1.503993	0.109845
C	0.378611	0.610833	-0.259527
C	1.718116	0.821878	0.437154
C	2.752655	-0.220335	0.092097
O	2.690758	-1.009617	-0.813985
O	3.818743	-0.160155	0.925571
H	4.450904	-0.829230	0.627711
H	1.606049	0.851376	1.522250
H	2.151352	1.786737	0.160183
H	-0.228898	1.503278	-0.110226
H	0.547377	0.505998	-1.332758
H	-2.152294	-1.786969	-0.159710
H	-1.606720	-0.852649	-1.522457

Table S12: Cartesian coordinates for the equilibrium structure of conformer II of adipic acid (AA-II) optimized at the B3LYP-D4/def2-QZVP level of theory.

Atom	X	Y	Z
C	-1.545591	-0.823188	-0.625728
C	-1.579261	0.503025	0.085399
O	-1.122664	0.741143	1.175162
O	-2.230951	1.437634	-0.640182
H	-2.220183	2.256266	-0.125509
C	-0.760881	-1.918750	0.096256
H	-0.994731	-1.883271	1.161842
H	-1.132800	-2.877304	-0.269336
C	0.761597	-1.918816	-0.096130
C	1.546402	-0.823106	0.625496
C	1.579069	0.503323	-0.085295
O	1.123597	0.740963	-1.175639
O	2.229219	1.438474	0.640931
H	2.218336	2.257079	0.126219
H	2.589551	-1.125845	0.748182
H	1.163554	-0.649143	1.631037
H	0.995477	-1.883695	-1.161722
H	1.133481	-2.877264	0.269786
H	-2.588464	-1.126269	-0.749704
H	-1.161714	-0.648636	-1.630792

Table S13: Cartesian coordinates for the equilibrium structure of conformer III of adipic acid (AA-III) optimized at the B3LYP-D4/def2-QZVP level of theory.

Atom	X	Y	Z
C	-2.034746	-1.126117	-0.352259
C	-2.720990	0.191234	-0.084926
O	-2.460134	0.957075	0.805013
O	-3.721692	0.419628	-0.967794
H	-4.134248	1.259259	-0.720949
C	-0.706687	-1.276550	0.382034
H	-0.858594	-1.051726	1.438913
H	-0.394358	-2.321685	0.322211
C	0.393745	-0.381713	-0.185266
C	1.694105	-0.512744	0.595813
C	2.819965	0.312199	0.027742
O	2.789322	0.950187	-0.990825
O	3.923804	0.252595	0.812675
H	4.604070	0.793056	0.387378
H	2.037320	-1.550993	0.623227
H	1.559653	-0.222660	1.640078
H	0.071086	0.658998	-0.168491
H	0.574070	-0.632814	-1.232024
H	-2.738817	-1.904827	-0.046779
H	-1.915090	-1.241260	-1.430677

Table S14: Cartesian coordinates for the equilibrium structure of conformer IV of adipic acid (AA-IV) optimized at the B3LYP-D4/def2-QZVP level of theory.

Atom	X	Y	Z
C	-1.838323	-0.007394	-0.629228
C	-3.188722	-0.000732	0.039346
O	-3.393734	0.011634	1.224098
O	-4.193059	-0.010341	-0.870524
H	-5.024678	-0.004890	-0.376367
C	-0.675835	-0.002022	0.354385
H	-0.757296	0.872534	1.002622
H	-0.757644	-0.869092	1.012606
C	0.675719	-0.006286	-0.354498
C	1.838211	-0.001837	0.629116
C	3.188605	-0.001419	-0.039501
O	3.393617	0.006595	-1.224292
O	4.192946	-0.008360	0.870390
H	5.024562	-0.005453	0.376206
H	1.798725	-0.868099	1.294145
H	1.796430	0.869501	1.287607
H	0.757853	0.861087	-1.012195
H	0.756855	-0.880546	-1.003261
H	-1.798830	-0.880630	-1.285161
H	-1.796548	0.856938	-1.296799

Table S15: Cartesian coordinates for the equilibrium structure of conformer V of adipic acid (AA-V) optimized at the B3LYP-D4/def2-QZVP level of theory.

Atom	X	Y	Z
C	-1.399938	0.022302	-0.742110
C	-2.439977	0.129705	0.345127
O	-2.747217	-0.735964	1.121500
O	-3.038258	1.345995	0.345212
H	-3.714213	1.330383	1.037336
C	-0.439911	-1.152242	-0.566935
H	-1.021243	-2.071822	-0.503007
H	0.176111	-1.230591	-1.462556
C	0.465092	-1.068110	0.666927
C	1.381011	0.153028	0.710996
C	2.420615	0.177199	-0.381702
O	2.761775	-0.757234	-1.057677
O	2.974847	1.406860	-0.516205
H	3.653427	1.339523	-1.202632
H	1.931695	0.174595	1.655467
H	0.828609	1.092407	0.681525
H	1.080003	-1.967161	0.702390
H	-0.149481	-1.071054	1.566924
H	-1.951471	-0.081291	-1.680694
H	-0.882534	0.978677	-0.819882

Table S16: Cartesian coordinates for the equilibrium structure of conformer VI of adipic acid (AA-VI) optimized at the B3LYP-D4/def2-QZVP level of theory.

Atom	X	Y	Z
C	-1.752303	-1.128155	0.015698
C	-1.795330	0.375018	-0.101910
O	-1.143545	1.155412	0.540422
O	-2.706714	0.779409	-1.019366
H	-2.710551	1.746982	-1.007913
C	-0.482505	-1.674354	0.669044
H	-0.209999	-1.041297	1.511916
H	-0.708216	-2.660199	1.077344
C	0.710979	-1.822219	-0.280292
C	1.235566	-0.525781	-0.893549
C	1.832449	0.420806	0.118603
O	2.187628	0.128270	1.229591
O	1.975867	1.665597	-0.388278
H	2.359986	2.213270	0.310301
H	0.477502	0.008354	-1.464699
H	2.031293	-0.751136	-1.609171
H	0.439090	-2.497239	-1.095742
H	1.525034	-2.300441	0.264352
H	-2.627807	-1.394421	0.614858
H	-1.927634	-1.563589	-0.969266

Table S17: Cartesian coordinates for the equilibrium structure of conformer VII of adipic acid (AA-VII) optimized at the B3LYP-D4/def2-QZVP level of theory.

Atom	X	Y	Z
C	-1.707139	-0.438437	-1.069263
C	-2.770870	-0.263599	-0.016244
O	-3.340126	-1.147026	0.568344
O	-3.037403	1.046070	0.222798
H	-3.726750	1.073806	0.900644
C	-0.375482	0.232643	-0.707676
H	0.313072	0.082981	-1.538759
H	-0.529560	1.308086	-0.603741
C	0.242907	-0.327197	0.572862
C	1.577421	0.320030	0.925870
C	2.699542	-0.053759	-0.010974
O	2.669486	-0.919816	-0.845183
O	3.800465	0.704640	0.205709
H	4.484281	0.395910	-0.405127
H	1.900305	0.024883	1.927520
H	1.502397	1.408908	0.951371
H	0.381033	-1.404410	0.470817
H	-0.441442	-0.176377	1.410055
H	-1.574271	-1.507695	-1.218191
H	-2.087855	-0.011261	-1.999023

Table S18: Cartesian coordinates for the equilibrium structure of conformer VIII of adipic acid (AA-VIII) optimized at the B3LYP-D4/def2-QZVP level of theory.

Atom	X	Y	Z
C	-1.838879	-1.363213	0.223362
C	-2.807749	-0.622395	-0.661720
O	-3.181400	-0.971384	-1.750175
O	-3.235815	0.531954	-0.089352
H	-3.849989	0.944416	-0.712348
C	-0.579812	-0.551767	0.557404
H	-0.865009	0.347726	1.105826
H	0.040077	-1.153068	1.221968
C	0.226708	-0.167579	-0.682383
C	1.491488	0.617568	-0.353288
C	2.552959	-0.204257	0.335551
O	2.571211	-1.403184	0.430568
O	3.538097	0.577052	0.838667
H	4.194620	-0.012144	1.236069
H	1.275335	1.489602	0.266470
H	1.950813	1.010367	-1.264265
H	-0.387763	0.440876	-1.348900
H	0.494938	-1.067373	-1.237484
H	-2.360778	-1.619155	1.147300
H	-1.578755	-2.288073	-0.286613

Table S19: Cartesian coordinates for the equilibrium structure of conformer IX of adipic acid (AA-IX) optimized at the B3LYP-D4/def2-QZVP level of theory.

Atom	X	Y	Z
C	-1.374110	-0.504475	0.727546
C	-2.284580	0.384841	-0.074657
O	-2.139133	1.567764	-0.246879
O	-3.301946	-0.306933	-0.642155
H	-3.818570	0.322749	-1.164260
C	-0.444211	-1.339281	-0.174619
H	0.216212	-1.918417	0.469093
H	-1.047314	-2.055186	-0.733675
C	0.392690	-0.524484	-1.166054
C	1.259357	0.566246	-0.540580
C	2.329292	0.041587	0.381644
O	2.695763	-1.101854	0.467489
O	2.880508	1.032635	1.122233
H	3.577614	0.631672	1.660165
H	0.664175	1.307207	-0.007996
H	1.777819	1.125965	-1.323722
H	-0.262556	-0.048098	-1.897711
H	1.029944	-1.213735	-1.720142
H	-0.800923	0.134965	1.394486
H	-1.977431	-1.178272	1.334915

3.2 Conformers of adipic anhydride.

Table S20: Cartesian coordinates for the equilibrium structure of conformer I of adipic anhydride (AAD-I) optimized at the B3LYP-D4/def2-QZVP level of theory.

Atom	X	Y	Z
C	-1.274763	-0.625271	0.865956
H	-0.702758	-0.482935	1.785136
H	-2.319108	-0.769306	1.130757
C	-0.754898	-1.857187	0.108370
H	-1.269399	-1.926334	-0.852757
H	-1.034750	-2.746081	0.674197
C	0.756586	-1.856581	-0.108375
H	1.037159	-2.745278	-0.674155
H	1.271129	-1.925270	0.852763
C	1.275489	-0.624273	-0.866002
H	0.703524	-0.482539	-1.785295
H	2.320008	-0.767425	-1.130591
C	1.221715	0.642695	-0.044225
O	2.157220	1.143927	0.498140
O	-0.000571	1.291894	-0.000269
C	-1.222258	0.641640	0.044021
O	-2.158444	1.142298	-0.497705

Table S21: Cartesian coordinates for the equilibrium structure of conformer II of adipic anhydride (AAD-II) optimized at the B3LYP-D4/def2-QZVP level of theory.

Atom	X	Y	Z
C	-1.196803	-0.242171	-1.049547
H	-2.144546	-0.082485	-1.555608
H	-0.404897	-0.198890	-1.798247

Atom	X	Y	Z
C	-1.184673	-1.606627	-0.329995
H	-1.035720	-2.386704	-1.078015
H	-2.160380	-1.780427	0.122514
C	-0.103887	-1.694875	0.751045
H	0.001668	-2.729918	1.075736
H	-0.417371	-1.131627	1.631941
C	1.255498	-1.183200	0.285960
H	1.598623	-1.711102	-0.605914
H	2.015199	-1.370620	1.047309
C	1.372111	0.298946	-0.018861
O	2.362136	0.808924	-0.440148
O	0.299512	1.114264	0.329959
C	-1.007020	0.873440	-0.053840
O	-1.873837	1.543874	0.414364

3.3 Conformers of ε-caprolactone.

Table S22: Cartesian coordinates for the equilibrium structure of conformer I of ε-caprolactone (ε-CL-I) optimized at the B3LYP-D4/def2-QZVP level of theory.

Atom	X	Y	Z
C	1.952535	0.644677	0.067548
C	1.689643	-0.821868	0.406745
C	0.595293	-1.476665	-0.424375
H	0.685435	-1.212902	-1.480960

Atom	X	Y	Z
H	0.661209	-2.559003	-0.347694
O	-0.734668	-1.192166	0.040830
C	-1.288191	0.040539	-0.043569
O	-2.401572	0.198466	0.377604
C	-0.467237	1.166702	-0.638080
C	0.742873	1.564208	0.225526
H	1.036097	2.577633	-0.051301
H	0.435765	1.609977	1.273089
H	-1.152589	2.004267	-0.731990
H	-0.132942	0.900457	-1.643888
H	2.608385	-1.391648	0.249447
H	1.430854	-0.935533	1.462309
H	2.767588	1.015718	0.691716
H	2.307134	0.706809	-0.966170

Table S23: Cartesian coordinates for the equilibrium structure of conformer II of ε-caprolactone (ε-CL-II) optimized at the B3LYP-D4/def2-QZVP level of theory.

Atom	X	Y	Z
C	1.396797	0.691465	0.810621
C	1.829571	-0.659985	0.234402
C	0.742437	-1.337499	-0.596998
H	0.711739	-0.934745	-1.609204
H	0.921412	-2.406775	-0.677381
O	-0.554394	-1.229036	0.009451
C	-1.226207	-0.048485	-0.022679
O	-2.272160	0.038642	0.558350

Atom	X	Y	Z
C	-0.599336	1.088233	-0.808374
C	0.720029	1.629881	-0.192292
H	1.415319	1.875091	-0.997244
H	0.499064	2.566253	0.318924
H	-1.349164	1.871919	-0.854017
H	-0.429539	0.749940	-1.830800
H	2.707264	-0.536928	-0.405377
H	2.125549	-1.315916	1.053999
H	0.715762	0.518866	1.646466
H	2.268705	1.191486	1.234374

3.4 Isomers of the monohydrated ε-caprolactone.

Table S24: Cartesian coordinates for the equilibrium structure of isomer I of the monohydrated ε-caprolactone (ε-CL-1w-I) optimized at the B3LYP-D4/def2-QZVP level of theory.

Atom	X	Y	Z
C	-1.838389	1.523545	-0.079122
C	-0.314539	1.624608	-0.110197
H	-0.019239	2.612938	0.242213
H	0.053309	1.546134	-1.135602
C	0.403906	0.580717	0.764492
C	0.503327	-0.774321	0.102680
O	1.547254	-1.243574	-0.285616
O	-0.615910	-1.492634	-0.092854

Atom	X	Y	Z
C	-1.918768	-1.014462	0.293739
C	-2.400131	0.174010	-0.525645
H	-3.489717	0.201345	-0.452727
H	-2.164223	-0.016479	-1.575478
H	-2.558842	-1.876212	0.124675
H	-1.929476	-0.794797	1.363496
H	1.423010	0.899725	0.960037
H	-0.107427	0.487481	1.725645
H	-2.262282	2.311054	-0.704734
H	-2.182487	1.724965	0.940265
O	3.606707	0.675296	-0.135273
H	3.037475	-0.104725	-0.246820
H	4.503197	0.368875	-0.283899

Table S25: Cartesian coordinates for the equilibrium structure of isomer II of the monohydrated ε-caprolactone (ε-CL-1w-II) optimized at the B3LYP-D4/def2-QZVP level of theory.

Atom	X	Y	Z
C	2.073434	0.336605	-0.622427
C	1.032044	-0.576365	-1.267616
H	1.528796	-1.213308	-2.000470
H	0.295633	0.016431	-1.812036
C	0.300123	-1.491425	-0.266482
C	-0.791833	-0.766371	0.485310
O	-1.964926	-0.932888	0.248020
O	-0.455312	0.124869	1.431909
C	0.909814	0.529272	1.655395

Atom	X	Y	Z
C	1.526100	1.256967	0.468951
H	2.340781	1.875906	0.851590
H	0.776915	1.937320	0.058412
H	0.833555	1.192725	2.512618
H	1.507518	-0.335397	1.953363
H	-0.195727	-2.301076	-0.794352
H	1.016412	-1.933626	0.429918
H	2.541113	0.944414	-1.398869
H	2.870572	-0.279833	-0.193979
O	-1.923223	1.363071	-1.523556
H	-2.273109	0.670504	-0.946288
H	-2.636398	1.995792	-1.629629

3.5 Conformers of the 6-hydroxycaproic acid.

Table S26: Cartesian coordinates for the equilibrium structure of conformer I of 6-hydroxycaproic acid (6-HCA-I) optimized at the B3LYP-D4/def2-QZVP level of theory.

Atom	X	Y	Z
C	-1.880319	-0.325495	0.072463
O	-1.130658	-1.233833	-0.192748
O	-3.117120	-0.544572	0.562689
H	-3.234074	-1.502341	0.638348
C	-1.598587	1.145058	-0.109465
C	-0.161826	1.423313	-0.534873

Atom	X	Y	Z
H	0.060713	0.803741	-1.402922
H	-0.085157	2.458808	-0.870276
C	0.854192	1.162401	0.584471
H	0.874800	2.022481	1.257466
C	2.271849	0.865289	0.092085
C	2.378229	-0.413192	-0.743765
H	1.895742	-0.277883	-1.717576
H	3.429467	-0.625673	-0.941047
O	1.853634	-1.556158	-0.087481
H	0.890412	-1.534147	-0.144805
H	2.653413	1.700797	-0.503862
H	2.929468	0.768438	0.958338
H	0.526813	0.316225	1.189711
H	-1.853926	1.653305	0.823013
H	-2.315556	1.510628	-0.849028

Table S27: Cartesian coordinates for the equilibrium structure of conformer II of of 6-hydroxycaproic acid (6-HCA-II) optimized at the B3LYP-D4/def2-QZVP level of theory.

Atom	X	Y	Z
C	1.807787	0.282476	0.018239
O	1.076475	1.243734	0.034015
O	3.096098	0.367038	0.406771
H	3.263517	1.286349	0.658856
C	1.443399	-1.108807	-0.433344
C	-0.046604	-1.275049	-0.704690
H	-0.373776	-0.489482	-1.382291

Atom	X	Y	Z
H	-0.204054	-2.217759	-1.231295
C	-0.902020	-1.258290	0.573284
H	-0.895438	-2.258035	1.012781
C	-2.345836	-0.796172	0.342434
C	-2.516389	0.723756	0.353546
H	-3.567495	0.970378	0.197599
H	-2.236609	1.107504	1.342638
O	-1.796038	1.406511	-0.660601
H	-0.861299	1.440352	-0.418565
H	-2.719926	-1.197267	-0.603280
H	-2.991041	-1.197435	1.128886
H	-0.445134	-0.601837	1.318931
H	1.804900	-1.811454	0.321006
H	2.037701	-1.309207	-1.328759

Table S28: Cartesian coordinates for the equilibrium structure of conformer III of of 6-hydroxycaproic acid (6-HCA-III) optimized at the B3LYP-D4/def2-QZVP level of theory.

Atom	X	Y	Z
C	-1.645619	-0.337210	0.071067
O	-1.011186	-1.321629	0.365089
O	-2.808331	-0.426825	-0.608544
H	-2.981748	-1.366343	-0.762690
C	-1.321189	1.091276	0.422270
C	0.105995	1.364030	0.900322
H	0.078348	2.282345	1.489492
H	0.429237	0.572657	1.572269

Atom	X	Y	Z
C	1.133049	1.563631	-0.223146
H	0.790295	2.390778	-0.852126
C	1.430802	0.358125	-1.122692
C	2.326915	-0.713514	-0.501923
H	2.485886	-1.511868	-1.237788
H	3.305430	-0.281866	-0.280899
O	1.859431	-1.250716	0.724073
H	0.924576	-1.477088	0.632586
H	0.504365	-0.098492	-1.477941
H	1.942894	0.712976	-2.021576
H	2.070269	1.898240	0.228974
H	-2.036422	1.352289	1.208208
H	-1.589575	1.718934	-0.429551

4 Measured rotational transitions.

4.1 Frequency list of adipic acid.

Table S29: Assigned rotational transitions for conformer III of adipic acid (AA-III).

J'	K_a'	K_c'	J"	K_a"	K_c"	ν_{obs}/MHz	ν_{calc}/MHz	$\Delta\nu$/kHz
10	2	8	9	1	9	18019.264	18019.262	2.3
11	2	10	10	1	9	18076.536	18076.531	4.8
5	3	3	4	2	2	18643.174	18643.170	4.3
5	3	2	4	2	3	18644.269	18644.266	3.3

J'	K_a'	K_c'	J"	K_a"	K_c"	ν_{obs}/MHz	ν_{calc}/MHz	$\Delta\nu$/kHz
12	2	11	11	1	10	18904.364	18904.369	-5.0
11	2	9	10	1	10	19035.856	19035.857	-1.5
6	3	4	5	2	3	19560.185	19560.180	4.5
6	3	3	5	2	4	19562.741	19562.741	-0.1
4	4	1	4	3	2	19674.997	19675.003	-6.1
4	4	0	4	3	1	19674.997	19675.002	-4.7
5	4	1	5	3	2	19674.997	19674.991	5.6
5	4	2	5	3	3	19674.997	19674.997	0.0
13	2	12	12	1	11	19724.291	19724.290	1.0
7	3	5	6	2	4	20476.471	20476.468	2.7
7	3	4	6	2	5	20481.598	20481.597	1.4
8	3	6	7	2	5	21391.719	21391.728	-9.3
8	3	5	7	2	6	21400.979	21400.973	6.4
9	3	7	8	2	6	22305.610	22305.604	6.0
9	3	6	8	2	7	22321.010	22321.033	-23.4
10	3	8	9	2	7	23217.682	23217.688	-5.8
10	3	7	9	2	7	23218.032	23218.030	1.6
4	4	0	3	3	1	23346.605	23346.622	-17.2
4	4	1	3	3	0	23346.605	23346.622	-17.0
11	3	9	10	2	8	24127.511	24127.523	-11.7
11	3	8	10	2	9	24164.000	24163.992	8.4
5	4	1	4	3	2	24264.590	24264.577	12.6
5	4	2	4	3	1	24264.590	24264.576	14.1
12	3	10	11	2	9	25034.615	25034.605	10.0
12	3	9	11	2	10	25087.345	25087.345	0.0
6	4	2	5	3	3	25182.527	25182.524	2.8

J'	K_a'	K_c'	J"	K_a"	K_c"	ν_{obs}/MHz	ν_{calc}/MHz	$\Delta\nu$/kHz
6	4	3	5	3	2	25182.527	25182.519	8.4

4.2 Frequency list of adipic anhydride.

Table S30: Assigned rotational transitions for conformer I of adipic anhydride (AAD-I).

J'	K_a'	K_c'	J"	K_a"	K_c"	ν_{obs}/MHz	ν_{calc}/MHz	$\Delta\nu$/kHz
6	3	4	5	2	3	18229.394	18229.397	-2.6
4	2	2	3	1	3	18241.800	18241.798	1.5
7	1	6	6	2	5	18283.646	18283.649	-2.9
7	2	6	6	1	5	18304.802	18304.803	-1.0
5	4	2	4	3	1	19152.018	19152.017	1.3
7	3	4	6	4	3	19932.021	19932.018	3.1
4	3	2	3	0	3	19987.791	19987.784	6.6
7	3	5	6	2	4	20125.268	20125.267	0.6
5	4	1	4	3	2	20258.214	20258.215	-0.9
8	1	7	7	2	6	20563.841	20563.844	-2.9
8	2	7	7	1	6	20568.260	20568.263	-3.0
5	5	1	4	4	0	21005.037	21005.040	-3.4
5	5	0	4	4	1	21066.354	21066.353	0.8
6	4	3	5	3	2	21202.247	21202.244	3.3
9	1	9	8	0	8	21258.806	21258.810	-3.5
9	0	9	8	1	8	21258.806	21258.800	6.0
4	4	1	3	1	2	21278.672	21278.673	-0.6
5	3	2	4	2	3	21340.067	21340.070	-3.2

J'	K_a'	K_c'	J"	K_a"	K_c"	ν_{obs}/MHz	ν_{calc}/MHz	$\Delta\nu$/kHz
8	2	6	7	3	5	22108.690	22108.687	3.4
8	4	4	7	5	3	22149.771	22149.767	3.8
8	3	6	7	2	5	22229.342	22229.338	3.8
9	1	8	8	2	7	22838.578	22838.576	1.5
9	2	8	8	1	7	22839.445	22839.439	6.0
7	4	4	6	3	3	22885.714	22885.707	6.8
8	3	5	7	4	4	23150.561	23150.562	-0.8
10	1	10	9	0	9	23533.064	23533.074	-9.8
10	0	10	9	1	9	23533.064	23533.072	-8.3
6	5	2	5	4	1	23774.730	23774.732	-2.3
5	2	3	4	1	4	23940.639	23940.641	-2.0
6	5	1	5	4	2	24265.107	24265.106	1.0
9	2	7	8	3	6	24417.398	24417.403	-5.4
9	3	7	8	2	6	24447.156	24447.151	4.9
8	4	5	7	3	4	24490.605	24490.603	2.2
6	4	2	5	3	3	24521.084	24521.091	-7.1
5	4	2	4	1	3	24714.711	24714.720	-9.3
5	3	3	4	0	4	24824.049	24824.042	7.3
10	1	9	9	2	8	25112.390	25112.400	-9.7
10	2	9	9	1	8	25112.560	25112.560	0.2
6	6	1	5	5	0	25368.934	25368.931	3.0
6	6	0	5	5	1	25387.352	25387.351	1.4
11	0	11	10	1	10	25807.337	25807.325	11.8
11	1	11	10	0	10	25807.337	25807.329	7.6
9	3	6	8	4	5	25839.099	25839.105	-6.3

Table S31: Assigned rotational transitions for conformer II of andipic anhydride (AAD-II).

J'	K_a'	K_c'	J"	K_a"	K_c"	ν_{obs}/MHz	ν_{calc}/MHz	$\Delta\nu$/kHz
5	2	3	4	1	3	18588.716	18588.717	-0.9
5	3	2	4	2	2	18616.865	18616.869	-4.1
7	1	6	6	2	5	18712.134	18712.132	1.6
7	2	6	6	1	5	18766.529	18766.528	1.1
6	3	4	5	2	3	18878.567	18878.565	1.7
5	2	4	4	1	4	19301.598	19301.597	1.2
7	3	4	6	4	3	19347.670	19347.679	-8.7
8	0	8	7	1	7	19548.231	19548.227	4.4
8	1	8	7	0	7	19548.459	19548.453	5.7
5	4	2	4	3	1	19758.685	19758.682	2.7
7	2	5	6	3	4	19893.778	19893.781	-3.3
5	4	1	4	3	1	19967.137	19967.136	1.2
4	3	2	3	0	3	20249.037	20249.034	3.2
5	4	2	4	3	2	20297.724	20297.725	-0.9
5	4	1	4	3	2	20506.179	20506.178	0.6
7	3	5	6	2	4	20741.452	20741.455	-3.0
8	4	4	7	5	3	20970.241	20970.233	8.5
5	3	2	4	2	3	21069.257	21069.258	-0.9
8	1	7	7	2	6	21076.357	21076.358	-0.9
8	2	7	7	1	6	21090.205	21090.210	-5.0
5	5	1	4	4	0	21605.149	21605.143	5.9
5	5	0	4	4	0	21611.823	21611.825	-1.7
5	5	1	4	4	1	21631.327	21631.328	-1.1
5	5	0	4	4	1	21638.018	21638.010	8.3

J'	K_a'	K_c'	J"	K_a"	K_c"	ν_{obs}/MHz	ν_{calc}/MHz	$\Delta\nu$/kHz
9	0	9	8	1	8	21896.020	21896.027	-7.0
9	1	9	8	0	8	21896.070	21896.072	-2.2
6	4	3	5	3	2	21983.610	21983.611	-0.7
6	3	3	5	2	3	22103.581	22103.579	2.2
4	4	1	3	1	2	22202.286	22202.295	-9.2
8	2	6	7	3	5	22501.528	22501.533	-4.8
6	2	4	5	1	4	22654.278	22654.281	-3.2
8	3	5	7	4	4	22974.806	22974.808	-1.7
6	1	5	5	0	5	23108.904	23108.895	9.4
6	3	4	5	2	4	23124.355	23124.344	10.8
6	2	5	5	1	5	23147.889	23147.886	3.3
9	1	8	8	2	7	23427.342	23427.344	-2.1
9	2	8	8	1	7	23430.642	23430.637	5.0
5	2	3	4	1	4	23547.367	23547.376	-8.8
6	4	3	5	3	3	23570.540	23570.544	-3.8
7	4	4	6	3	3	23810.046	23810.042	3.6
10	1	10	9	0	9	24243.762	24243.770	-8.5
10	0	10	9	1	9	24243.762	24243.762	0.3
6	4	2	5	3	3	24395.473	24395.475	-2.0
6	5	2	5	4	1	24461.841	24461.838	3.4
6	5	1	5	4	2	24737.289	24737.292	-2.9
5	3	3	4	0	4	24891.248	24891.248	0.1
9	2	7	8	3	6	24937.091	24937.100	-9.5
9	3	7	8	2	6	25027.761	25027.764	-2.7
5	4	2	4	1	3	25383.676	25383.677	-0.9
8	4	5	7	3	4	25438.737	25438.739	-2.4

J'	K_a'	K_c'	J"	K_a"	K_c"	ν_{obs}/MHz	ν_{calc}/MHz	$\Delta\nu$/kHz
10	1	9	9	2	8	25775.150	25775.145	4.8
10	2	9	9	1	8	25775.900	25775.890	10.2

4.3 Frequency list of the monohydrated ε-caprolactone.

Table S32: Assigned rotational transitions for the monohydrated ε-caprolactone (ε-CL-$H_2{}^{16}O$-I).

J'	K_a'	K_c'	J"	K_a"	K_c"	ν_{obs}/MHz	ν_{calc}/MHz	$\Delta\nu$/kHz
4	4	1	3	3	0	18192.473	18192.479	-6.2
4	4	0	3	3	1	18194.376	18194.388	-12.1
10	4	6	9	4	5	18294.462	18294.463	-1.3
10	2	8	9	2	7	18813.161	18813.161	0.3
10	3	7	9	3	6	18821.029	18821.029	-0.4
11	2	10	10	2	9	18887.330	18887.329	0.6
6	3	3	5	2	4	19066.924	19066.917	6.6
11	1	10	10	1	9	19133.724	19133.729	-4.7
12	1	12	11	1	11	19268.829	19268.832	-3.1
12	0	12	11	0	11	19276.562	19276.572	-9.6
11	3	9	10	3	8	19699.143	19699.141	1.8
5	4	2	4	3	1	19981.104	19981.106	-2.1
5	4	1	4	3	1	19982.036	19982.039	-3.0
5	4	2	4	3	2	19993.650	19993.646	4.3
5	4	1	4	3	2	19994.579	19994.579	0.4
11	4	8	10	4	7	19971.244	19971.260	-15.7

J'	K_a'	K_c'	J"	K_a"	K_c"	ν_{obs}/MHz	ν_{calc}/MHz	$\Delta\nu$/kHz
11	5	6	10	5	5	19966.657	19966.674	-17.4
11	4	7	10	4	6	20253.026	20253.036	-9.6
12	2	11	11	2	10	20477.605	20477.610	-5.1
11	2	9	10	2	8	20530.312	20530.311	1.0
8	3	6	7	2	5	20639.916	20639.922	-5.9
12	1	11	11	1	10	20640.936	20640.933	3.4
11	3	8	10	3	7	20819.711	20819.699	12.5
13	1	13	12	1	12	20822.018	20822.018	-0.4
13	0	13	12	0	12	20826.151	20826.149	1.8
7	3	4	6	2	5	21291.120	21291.122	-1.8
12	3	10	11	3	9	21401.395	21401.388	7.1
6	4	3	5	3	2	21748.030	21748.029	0.6
6	4	2	5	3	2	21752.640	21752.653	-12.6
12	4	9	11	4	8	21789.240	21789.237	2.6
12	5	8	11	5	7	21792.220	21792.238	-18.3
6	4	2	5	3	3	21802.088	21802.092	-4.0
6	4	3	5	3	3	21797.461	21797.469	-7.8
10	6	5	9	6	4	18047.602	18047.601	1.4
10	6	4	9	6	3	18047.970	18047.952	18.4
10	5	6	9	5	5	18097.582	18097.571	11.2
10	5	5	9	5	4	18107.782	18107.772	10.2
6	3	4	5	2	3	18111.314	18111.322	-8.3
10	4	7	9	4	6	18141.481	18141.479	1.9
6	3	3	5	2	3	18255.916	18255.912	3.8
10	2	9	9	1	8	18284.566	18284.563	3.4
6	2	4	5	1	5	18668.269	18668.276	-7.0

J'	K_a'	K_c'	J"	K_a"	K_c"	ν_{obs}/MHz	ν_{calc}/MHz	$\Delta\nu$/kHz
6	3	4	5	2	4	18922.334	18922.328	6.4
7	3	5	6	2	4	19453.281	19453.278	3.0
11	5	7	10	5	6	19941.945	19941.961	-16.3
12	5	7	11	5	6	21846.381	21846.396	-14.8
13	2	12	12	2	11	22054.214	22054.216	-2.0
13	1	12	12	1	11	22156.965	22156.956	8.5
12	2	10	11	2	9	22165.407	22165.404	2.8
12	4	8	11	4	7	22259.912	22259.896	16.1
12	3	9	11	3	8	22771.769	22771.760	8.8
13	3	11	12	3	10	23074.409	23074.408	1.2
7	4	4	6	3	3	23468.048	23468.043	5.1
7	4	3	6	3	3	23484.773	23484.760	13.3
7	4	4	6	3	4	23612.645	23612.633	12.3
14	2	13	13	2	12	23620.892	23620.878	13.6
7	4	3	6	3	4	23629.361	23629.350	11.4
13	2	11	12	2	10	23725.724	23725.736	-12.3

Table S33: Assigned rotational transitions for the monohydrated ε-caprolactone (ε-CL-$H_2{}^{18}O$-I).

J'	K_a'	K_c'	J"	K_a"	K_c"	ν_{obs}/MHz	ν_{calc}/MHz	$\Delta\nu$/kHz
11	2	10	10	2	9	18134.256	18134.258	-1.7
11	1	10	10	1	9	18423.573	18423.570	3.4
12	0	12	11	1	11	18509.105	18509.094	10.7
12	1	12	11	1	11	18522.218	18522.219	-1.0

J'	K_a'	K_c'	J"	K_a"	K_c"	ν_{obs}/MHz	ν_{calc}/MHz	$\Delta\nu$/kHz
12	0	12	11	0	11	18533.018	18533.012	5.7
12	1	12	11	0	11	18546.125	18546.137	-12.0
6	3	3	5	2	4	18651.429	18651.434	-5.3
8	2	6	7	1	6	18775.136	18775.129	7.1
11	3	9	10	3	8	18855.302	18855.305	-3.0
11	5	7	10	5	6	19028.734	19028.724	10.1
11	5	6	10	5	5	19044.164	19044.154	10.0
11	4	8	10	4	7	19065.636	19065.639	-2.6
7	3	5	6	2	4	19175.251	19175.261	-9.8
11	4	7	10	4	6	19266.281	19266.282	-1.0
12	1	11	11	2	10	19323.268	19323.277	-9.4
12	2	11	11	2	10	19669.594	19669.589	5.1
11	2	9	10	2	8	19673.445	19673.454	-9.3
5	4	2	4	3	1	19782.226	19782.232	-5.8
5	4	1	4	3	2	19792.298	19792.311	-12.7
11	3	8	10	3	7	19803.143	19803.142	0.8
12	1	11	11	1	10	19872.178	19872.180	-2.2
13	1	13	12	1	12	20015.686	20015.685	0.7
13	0	13	12	0	12	20021.686	20021.688	-1.7
8	3	6	7	2	5	20350.143	20350.130	12.6
7	3	5	6	2	5	20452.070	20452.073	-3.1
12	3	10	11	3	9	20499.534	20499.534	-0.1
12	5	8	11	5	7	20791.243	20791.245	-1.9
12	4	9	11	4	8	20807.918	20807.924	-5.6
12	5	7	11	5	6	20825.333	20825.328	5.1
12	4	8	11	4	7	21151.293	21151.297	-4.1

J'	K_a'	K_c'	J"	K_a"	K_c"	ν_{obs}/MHz	ν_{calc}/MHz	$\Delta\nu$/kHz
13	2	12	12	2	11	21190.869	21190.875	-6.5
12	2	10	11	2	9	21283.008	21283.009	-0.8
13	1	12	12	1	11	21325.088	21325.089	-1.4
9	3	7	8	2	6	21393.666	21393.664	2.1
6	4	3	5	3	2	21475.586	21475.584	2.5
14	1	14	13	1	13	21507.866	21507.865	1.3
14	0	14	13	0	13	21511.159	21511.157	1.7
6	4	2	5	3	3	21516.074	21516.066	8.0
12	3	9	11	3	8	21688.610	21688.613	-2.7
13	3	11	12	3	10	22118.214	22118.210	3.5
13	6	8	12	6	7	22482.181	22482.181	0.4
13	6	7	12	6	6	22486.499	22486.497	2.3
13	4	10	12	4	9	22537.445	22537.445	0.2
13	5	9	12	5	8	22557.896	22557.912	-15.6
13	5	8	12	5	7	22627.137	22627.152	-14.7
14	2	13	13	2	12	22701.421	22701.414	6.7
14	1	13	13	1	12	22786.647	22786.643	4.3
13	2	11	12	2	10	22821.754	22821.754	0.5
15	1	15	14	1	14	22999.126	22999.128	-2.4
15	0	15	14	0	14	23000.911	23000.915	-3.6
13	4	9	12	4	8	23076.539	23076.530	9.2
11	3	9	10	2	8	23192.425	23192.429	-4.4
7	4	4	6	3	4	23242.210	23242.212	-2.1
7	4	3	6	3	4	23253.642	23253.630	12.4
13	3	10	12	3	9	23525.392	23525.381	11.4
14	3	12	13	3	11	23711.575	23711.570	5.0

J'	K_a'	K_c'	J"	K_a"	K_c"	ν_{obs}/MHz	ν_{calc}/MHz	$\Delta\nu$/kHz
15	2	14	14	2	13	24204.135	24204.132	3.2
14	6	9	13	6	8	24245.692	24245.694	-2.2

4.4 Frequency list of 6-hydroxycaproic acid.

Table S34: Assigned rotational transitions for 6-hydroxycaproic acid (6-HCA-I).

J'	K_a'	K_c'	J"	K_a"	K_c"	ν_{obs}/MHz	ν_{calc}/MHz	$\Delta\nu$/kHz
6	3	4	5	2	3	19176.889	19176.887	2.3
6	3	3	5	2	4	19667.840	19667.832	8.3
4	4	1	3	3	0	19763.293	19763.292	1.1
4	4	0	3	3	1	19763.930	19763.925	5.4
5	4	2	4	3	1	21461.129	21461.133	-4.0
5	4	1	4	3	2	21465.590	21465.595	-4.9
7	3	4	6	2	5	21572.853	21572.858	-5.5
8	3	6	7	2	5	21916.401	21916.402	-1.1
6	4	3	5	3	2	23150.576	23150.588	-12.3
6	4	2	5	3	3	23168.538	23168.539	-0.8
7	4	4	6	3	3	24822.005	24822.003	2.4
7	4	3	6	3	4	24876.002	24875.994	8.0
5	5	0	4	4	1	25167.128	25167.138	-10.3
5	5	1	4	4	0	25167.128	25167.114	13.7
11	2	10	10	2	9	18238.567	18238.567	-0.2
11	1	10	10	1	9	18672.255	18672.255	0.1
